# Knowledge, Attitude, and Practice Towards Antibiotics Use Among Medical Sector Final-Year Students in Egypt

**DOI:** 10.1007/s40670-024-02117-6

**Published:** 2024-08-02

**Authors:** Nourhan M. Emera, Iman A. El-Baraky, Maggie M. Abbassi, Nirmeen A. Sabry

**Affiliations:** Faculty of Pharmacy, Clinical Pharmacy Department, Cairo University, Kasr El Einy Street, Cairo, 11562 Egypt

**Keywords:** Antibiotic use, Antibiotic resistance, Knowledge, Attitude, Practice, Students

## Abstract

**Introduction:**

Medical sector students must be well-educated and competent to spread public awareness of antibiotics among the public to combat antibiotic resistance. This study aimed to assess the knowledge, attitude, and practice (KAP) of students regarding antibiotic use and resistance in Egypt.

**Methodology:**

A cross-sectional questionnaire was specially designed and self-administered by final-year students (medicine (MS), pharmacy (PS), dentistry (DS), and nursing (NS)) during the last semester at nine universities.

**Results:**

Among 1250 recruited students, with an 89% response rate, PS and MS showed the highest knowledge level, whereas NS scored the lowest. The study revealed some misconceptions and malpractices among students. Two-thirds of PS and NS, half of DS, and a third of MS believed antibiotics treat sore throats. Sixty percent of NS and DS were unaware that vancomycin treats methicillin-resistant *Staphylococcus aureus*. Over half of MS and NS and a third of DS and PS lacked knowledge of amoxicillin safety during pregnancy and breastfeeding. The prevalence of antibiotics self-medication (ABSM) was highest among PS (30%), followed by NS (27%) and DS (25%), while MS reported the lowest rate (16.6%). One-third of students preferred to use newer and more expensive antibiotics. Seven percent of the students used the local guidelines, 12% used the international guidelines as sources of information, and only 8% received relevant formal training.

**Conclusions:**

The study found misconceptions and injudicious antibiotic use among medical sector students. Effective educational interventions and relevant training are needed to enhance their KAP on rational antibiotic use to minimize antibiotic resistance.

**Supplementary Information:**

The online version contains supplementary material available at 10.1007/s40670-024-02117-6.

## Introduction

Antibiotics play an essential role in reducing mortality due to infections [1]. However, the indiscriminate and imprudent use of antibiotics has led to the emergence of antibiotic resistance, which is now a serious global health concern [2–4]. Antibiotic resistance contributes to increased rates of morbidity, mortality, and economic burden [5, 6], making it a priority issue for the World Health Organization (WHO) [7]. The WHO conducted online surveys about antibiotic resistance in developing countries, where Egypt showed a low level of awareness about prudent antibiotic use and a high frequency of inappropriate antibiotic use among the public [8]. This is exacerbated by factors such as antibiotic self-medication (ABSM), the easy availability of non-prescribed antibiotics in community pharmacies, high medical consultation costs, and inadequate awareness about antibiotic use [9]. Therefore, there is an increasing consensus that new plans and strategies for preventing antibiotic resistance should be developed [10].

Medical sector students, as future healthcare providers (HCPs), play a vital role in combating antibiotic resistance by promoting public awareness through disseminating public health education about the judicious use of antibiotics and how to minimize resistance toward them [11]. To competently undertake these responsibilities, undergraduate medical sector students should be well-educated about all the related principles of microbiology, pharmacology, and infectious diseases and receive the appropriate training about antibiotic use [12]. They should also be able to handle patients’ requests for antibiotics [13]. Therefore, it is essential to provide adequate undergraduate education and training on antibiotics for medical sector students [14].

This cross-sectional study aimed to investigate and compare the knowledge, attitude, and practice (KAP) related to antibiotic use and antibiotic resistance among final-year medical sector undergraduate students in Egypt. The study focused on students in four medical specialties: medicine, pharmacy, dentistry, and nursing. The main objective of the study was to evaluate the KAP levels among the students in these specialties that could be useful for implementing more effective and suitable antibiotic control intervention strategies to mitigate antibiotic resistance.

## Materials and Methodology

### Study Setting and Design

This was a descriptive cross-sectional questionnaire-based study among medical sector students conducted during the last semester of the final academic year 2019–2020. The study protocol was approved by the Research Ethics Committee at the Faculty of Pharmacy, Cairo University, Egypt (CL:2553). The students were stratified into four groups based on their specialties: medicine students (MS), pharmacy students (PS), dentistry students (DS), and nursing students (NS).

### Study Sample

The sample size was calculated using the Rao-soft online sample size calculator (Rao soft Inc., Seattle, WA). The calculated sample was 373, with a confidence level of 95%, a margin of error of 5%, and a response distribution of 50%. During the data collection period, a total of 1250 students participated, reducing the margin of error to 2.5%.

### Data Collection Tools and Instruments

The data were collected using a questionnaire designed and developed by the research team, with some of the questions adopted from different published questionnaires [13–22]. The questions were modified to suit the target population and to ensure comprehensibility. A preliminary questionnaire was piloted among 62 students in the medical sector to assess its content, length, and comprehension. All necessary modifications were made to shape the final form of the questionnaire based on a pilot study. Additionally, the research team took into consideration the antibiotic use culture within the Egyptian community.

To evaluate the survey’s test–retest reliability [23], the students participating were asked to answer the survey questionnaire twice during the psychometric validation. Internal consistency was assessed using Cronbach’s alpha correlation coefficient, which yielded a value of 0.74, indicating acceptable reliability and the validity of the scale questions [24].

Furthermore, the questionnaire underwent evaluation for face and content validity. Face validity ensured that the questionnaire’s appearance aligned with the study’s objectives, detecting any potential reluctance or ambiguity in questions [25].Content validity focused on ensuring the clarity, completeness, and comprehensiveness of questions and answers for their intended purpose [24, 26].

The final questionnaire consisted of five parts as shown in Appendix 1. The first part covered students’ demographic data and characteristics such as specialty and university name. The second part was composed of 18 “true/false/do not know” questions to examine the level of the students’ knowledge about antibiotics.

The third section consisted of 13 “yes/no/unsure” questions to assess the students’ attitudes regarding antibiotic use and resistance. The fourth part included eight questions that were stated as yes/no/unsure questions or multiple-choice types to evaluate students’ practice related to ABSM and antibiotic uses. The fifth part enquired about the sources of information and topics taught in the academic curricula about antibiotics among medical sector students; it contained five questions that were stated as yes/no/unsure questions or multiple-choice types.

This self-administered questionnaire was designed to be completed in less than 30 min and presented in English. The researcher explained the aim of the study to the participating students and requested them to fill out the questionnaire without using any resources.

### Data Analysis and Presentation

Descriptive statistics were used to generate frequencies, percentages, and proportions, which are presented in tables and figures. Statistical analyses were performed using the IBM Statistical Packages for Social Sciences (SPSS) version 22. Charts and figures were generated using Microsoft Excel 2010. For the analysis of knowledge scores, a scoring system was adopted to quantify the level of knowledge; each correct response was worth 1 point, while an incorrect or unsure response was worth 0 points. The normality of distribution was assessed using the Kolmogorov–Smirnov test. Because the scores were not normally distributed, the Kruskal–Wallis *H*-test was used, for non-normally distributed quantitative variables, to test the difference in the score among the four specialties students. Chi-square Fisher exact analysis, for categorical data, was used to compare the KAP responses among the four groups. The significance level of all the hypotheses was set at 0.05.

## Results

This self-administered questionnaire was conducted in 25 faculties belonging to nine universities, including private and public universities (Appendix 2).

### Demographic Data of the Students

The KAP questionnaire was completed by 1250 final-year students pursuing four specialties: MS (*n* = 217), PS (*n* = 388), DS (*n* = 291), and NS (*n* = 354). The response rate was 89%. It is worth noting that there was a gradual decrease in the percentage of male students as we moved from MS to PS to DS to NS. A detailed overview of the participants’ characteristics can be found in Table 1.

**Table 1:** Demographic data and characteristics of students (*N* = 1250)

Parameter	Number	Students’ specialties (field of study)			
Demographic characteristics	Total (*N* = 1250)	Medicine (*N* = 217)	Pharmacy (*N* = 388)	Dentistry (*N* = 291)	Nursing (*N* = 354)
Mean age (years) ± SD	22.68 ± 1.11	24.01 ± 0.96	22.51 ± 0.66	22.77 ± 0.81	21.99 ± 1.09
Gender (number (%) of males)	518 (41.4%)	123 (56.7%)	163 (42%)	118 (40.5%)	114 (32.2%)
The number (%) of students belonged to public universities	714 (57.1%)	217 (70.7%)	175 (45.1%)	154 (52.9%)	212 (59.9%)

*N*, number of students; *SD*, standard deviation

### Knowledge About Antibiotics and Their Resistance

According to the analysis of knowledge scores among different specialties, students revealed that MS and PS demonstrated the highest level of knowledge, with a median score of 85.3% (IQR = 76.5–91.2) for MS and 85.3% (IQR = 79.4–91.2) for PS. DS scored slightly lower, with a median score of 79.4% (IQR = 69.1–88.2). On the other hand, NS exhibited the lowest level of knowledge, with a median score of 69% (IQR = 61.8–76.5).

To further explore the differences in antibiotic knowledge between the four specialties, Table 2 presents the percentages and frequencies of correct answers to some of the significant questions.

**Table 2:** Students’ median scores (%) and students’ responses (expressed as frequency (%) of correct answers) to questions related to knowledge about antibiotic use and antibiotic resistance

Parameter	Total (*N* = 1250)	MS (*N* = 217)	PS (*N* = 388)	DS (*N* = 291)	NS (*N* = 354)	*P*
Students’ median knowledge scores about antibiotics (%)						
Median % (IQR)	79.4 (70.6–88.2)	85.3 (76.5–91.2)	85.3 (79.4–91.2)	79.4 (69.1–88.2)	67.7 (61.8–76.5)	< 0.001^a^
Pairwise comparisons (*P*)	*P*_MP_ = 0.613	*P*_MD_ < 0.001^a^	*P*_MN_ < 0.001^a^	*P*_PD_ < 0.001^a^	*P*_PN_ < 0.001^a^	*P*_DN_ < 0.001^a^
Students’ responses (*N* (%) of correct answers) to questions related to knowledge about antibiotic use and antibiotic resistance						
Antibiotics are used for sore throat	508 (40.6)	134 (61.8)	129 (33.2)	142 (48.8)	103 (29.1)	< 0.001^a^
Pairwise comparisons (*P*)	*P*_MP_ < 0.001^a^	*P*_MD_ = 0.004^a^	*P*_MN_ < 0.001^a^	*P*_PD_ < 0.001^a^	*P*_PN_ = 0.223	*P*_DN_ < 0.001^a^
The use of broad-spectrum antibiotics is better than narrow-spectrum ones	812 (65.0(	185 (85.3)	267 (68.8)	199 (68.4)	161 (45.5)	< 0.001^a^
Pairwise comparisons (*P*)	*P*_MP_ < 0.001^a^	*P*_MD_ = 0.001^a^	*P*_MN_ < 0.001^a^	*P*_PD_ = 0.905	*P*_PN_ < 0.001^a^	*P*_DN_ < 0.001^a^
Antibiotics are considered anti-inflammatory medications	972 (77.8)	194 (89.4)	341 (87.9)	233 (80.1)	204 (57.6)	< 0.001^a^
Pairwise comparisons (*P*)	*P*_MP_ = 0.577	*P*_MD_ = 0.004^a^	*P*_MN_ < 0.012^a^	*P*_PD_ < 0.005^a^	*P*_PN_ < 0.001^a^	*P*_DN_ < 0.001^a^
Antibiotics have antipyretic effects	825 (66.0)	159 (73.3)	276 (71.1)	202 (69.4)	188 (53.1)	< 0.001^a^
Pairwise comparisons (*P*)	*P*_MP_ = 0.575	*P*_MD_ = 0.349	*P*_MN_ < 0.001^a^	*P*_PD_ = 0.627	*P*_PN_ < 0.001^a^	*P*_DN_ < 0.001^a^
MRSA is susceptible to vancomycin	733 (58.6)	160 (73.7)	317 (81.7)	119 (40.9)	137 (38.7)	< 0.001^a^
Pairwise comparisons (*P*)	*P*_MP_ = 0.021^a^	*P*_MD_ < 0.001^a^	*P*_MN_ < 0.001^a^	*P*_PD_ < 0.001^a^	*P*_PN_ < 0.001^a^	*P*_DN_ = 0.571
Amoxicillin is considered safe to use during the first trimester of pregnancy and breastfeeding	645 (51.6)	106 (48.8)	242 (62.4)	174 (59.8)	123 (34.7)	< 0.001^a^
Pairwise comparisons (P)	P_MP_ < 0.001^a^	P_MD_ = 0.014^a^	P_MN_ < 0.001^a^	P_PD_ = 0.495	P_PN_ < 0.001	P_DN_ < 0.001^a^
Antibiotic usage disturbs the gut flora and causes diarrhea and super-infection	952 (76.2)	197 (90.8)	339 (87.4)	223 (76.6)	193 (54.5)	< 0.001^a^
Pairwise comparisons (*P*)	*P*_MP_ = 0.205	*P*_MD_ = 0.001^a^	*P*_MN_ < 0.001^a^	*P*_PD_ < 0.001^a^	*P*_PN_ < 0.001^a^	*P*_DN_ < 0.001^a^
Tetracycline could be harmful to a child’s teeth	975 (78.0)	180 (82.9)	367 (94.6)	280 (96.2)	148 (41.8)	< 0.001^a^
Pairwise comparisons (*P*)	*P*_MP_ < 0.00^a^	*P*_MD_ = 0.004^a^	*P*_MN_ < 0.001^a^	*P*_PD_ = 0.321	*P*_PN_ < 0.001^a^	*P*_DN_ < 0.001^a^
Antibiotic resistance is due to excessive antibiotic use in animal food (cattle, sheep, poultry)	672 (53.8)	112 (51.6)	239 (61.6)	139 (47.8)	182 (51.4)	0.002^a^
Pairwise comparisons (*P*)	*P*_MP_ < 0.017^a^	*P*_MD_ = 0.391	*P*_MN_ = 0.963	*P*_PD_ < 0.001^a^	*P*_PN_ = 0.005^a^	*P*_DN_ = 0.357

Comparison between groups and pairwise comparisons using the Chi-square Fisher exact test for categorical variables and Kruskal–Wallis for non-normally distributed continuous variables (the *p*-value on the right column of the table)

*Abbreviation: MRSA*, methicillin-resistant *Staphylococcus aureus*; *MS*, medicine students; *PS*, pharmacy students; *DS*, dentistry students; *NS*, nursing students; *N*, number of students; *IQR*, interquartile range; *P*, *p*-value at level of significance < 0.05; *P*_*MP*_, *p*-value for comparing medicine and pharmacy; *P*_*MD*_, *p*-value for comparing medicine and dentistry; *P*_*MN*_, *p*-value for comparing medicine and nursing; *P*_*PD*_, *p*-value for comparing pharmacy and dentistry; *P*_*PN*_, *p*-value for comparing pharmacy and nursing; *P*_*DN*_, *p*-value for comparing dentistry and nursing

^a^Statistically significant

Forty-nine percent of DS and 61% of MS knew that antibiotics are not effective for sore throat, whereas NS and PS were the least knowledgeable at only 29% and 33%, respectively. The study also revealed that about 55% of NS thought that broad-spectrum antibiotics are more effective than narrow-spectrum antibiotics. However, this misconception was held by a small percentage of MS (less than 15%) followed by less than 30% of DS and PS.

In terms of differentiating antibiotics from other drugs, half of the NS were unable to differentiate between antibiotics from other drugs, such as anti-inflammatory and antipyretic drugs. In contrast, the majority of MS, PS, and DS demonstrated the ability to distinguish between antibiotics and other medications.

Regarding antibiotic selection, it is worth noting that only one-third of DS and NS identified vancomycin as the drug of choice to treat methicillin-resistant *Staphylococcus aureus* (MRSA), while most of the PS and MS did.

Moreover, concerning knowledge of the safety of antibiotics, about 60% of PS and DS were aware that amoxicillin is safe for use during pregnancy and breastfeeding, whereas the lowest level of awareness regarding this knowledge was observed among NS and MS at 34% and 48%, respectively.

In addition, almost all MS and PS, with 91% and 88% respectively, correctly stated antibiotics’ side effects such as diarrhea, and superinfection; however, only 54% of NS possessed this knowledge. Furthermore, NS exhibited the lowest level of awareness, with only 41% regarding the possible harm to a child’s teeth from tetracycline use. In comparison, almost all DS (96%), PS (95%), and MS (83%) knew that using tetracycline should be avoided with children. Additionally, two-thirds of PS and about one-half of MS, DS, and NS were aware that the overuse of antibiotics in animal food was a probable cause of antibiotic resistance.

Details of all knowledge results of the respondents’ students in the four medical specialties are shown in Appendix 3.

### Level of Attitude Towards Antibiotics and Their Resistance

The percentages and frequencies of attitude responses to some important statements have illustrated the differences among the four specialties which are revealed in Table 3.

**Table 3:** The attitude of the surveyed students about antibiotics and antibiotic resistance (presented as frequency (%))

Questions of attitudes towards antibiotic use and its resistance (response)	Total (*N* = 1250)	MS (*N* = 217)	PS (*N* = 388)	DS (*N* = 291)	NS (*N* = 354)	*P*
When you get a fever, antibiotics help you to get better faster (No)	686 (54.9)	141 (65.0)	234 (60.3)	147 (50.5)	164 (46.3)	< 0.001^a^
Pairwise comparisons (*P*)	*P*_MP_ = 0.106	*P*_MD_ = 0.002^a^	*P*_MN_ < 0.001^a^	*P*_PD_ < 0.036^a^	*P*_PN_ < 0.001^a^	*P*_DN_ = 0.028^a^
When you have a cold, you should take antibiotics to prevent getting a more serious illness (No)	974 (77.9)	176 (81.1)	331 (85.3)	243 (83.5)	224 (63.3)	< 0.001^a^
Pairwise comparisons (*P*)	*P*_MP_ = 0.398	*P*_MD_ = 0.0647	*P*_MN_ < 0.001	*P*_PD_ = 0.723	*P*_PN_ < 0.001^a^	*P*_DN_ < 0.001^a^
You select more expensive and newer antibiotics to provide more effective action and fewer side effects (No)	776 (62.1)	131 (60.4)	267 (68.8)	188 (64.6)	190 (53.7)	< 0.001^a^
Pairwise comparisons (*P*)	*P*_MP_ = 0.054	*P*_MD_ = 0.180	*P*_MN_ = 0.292	*P*_PD_ = 0.453	*P*_PN_ < 0.001^a^	*P*_DN_ = 0.003^a^
It is acceptable to skip one or two doses of antibiotics as long the whole course will be continued (No)	946 (75.7)	164 (75.6)	323 (83.2)	234 (80.4)	225 (63.6)	< 0.001^a^
Pairwise comparisons (*P*)	*P*_MP_ = 0.064	*P*_MD_ = 0.308	*P*_MN_ = 0.001^a^	*P*_PD_ = 0.607	*P*_PN_ < 0.001^a^	*P*_DN_ < 0.001^a^
You ask your physician for antibiotic allergy (Yes)	897 (71.8)	140 (64.5)	292 (75.3)	207 (71.1)	258 (72.9)	0.160
You contribute to the development of antibiotic resistance, whenever you take an antibiotic (Yes)	738 (59.0)	110 (50.7)	228 (58.8)	177 (60.8)	223 (63.0)	0.038^a^
Pairwise comparisons (*P*)	*P*_MP_ = 0.120	*P*_MD_ = 0.057	*P*_MN_ < 0.008^a^	*P*_PD_ = 0.258	*P*_PN_ < 0.071	*P*_DN_ = 0.806

Comparisons between groups and pairwise comparisons were used with the Chi-square Fisher exact test (the *p*-value on the right column of the table)

*Abbreviation: MS*, medicine students; *PS*, pharmacy students; *DS*, dentistry students; *NS*, nursing students; *N*, number of students; *P*, *p*-value at the level of significance < 0.05; *P*_*MP*_, *p*-value for comparing medicine and pharmacy; *P*_*MD*_, *p*-value for comparing medicine and dentistry; *P*_*MN*_, *p*-value for comparing medicine and nursing; *P*_*PD*_, *p*-value for comparing pharmacy and dentistry; *P*_*PN*_, *p*-value for comparing pharmacy and nursing; *P*_*DN*_, *p*-value for comparing dentistry and nursing

^a^Statistically significant

Concerning the difference in attitude toward antibiotic use in self-limiting diseases among four specialties students, the findings of this study indicated that approximately two-thirds of MS and PS expressed their disapproval of using antibiotics to help resolve fever faster. Moreover, less than one-half of DS and NS denied resorting to antibiotics for fever treatment. Furthermore, the study revealed that over one-third of NS concurred with the use of antibiotics for the common cold as a preventive measure against more severe illnesses. In contrast, approximately 85% of students from other specialties disagreed with this attitude.

Moreover, about two-fifths of MS, DS, and PS students preferred newer and more expensive antibiotics to provide more effective action and fewer side effects, followed by half of NS who held the same attitude.

In addition, 83% of PS, 81% of DS, 76% of MS, and 63% of NS declined to skip antibiotic doses while taking a certain course of antibiotics.

In terms of seeking an antibiotic allergy test, approximately more than one-third of MS admitted to not asking their physicians for such a test. This was followed by one-fourth of students from the other specialties who did not seek an antibiotic allergy test. Additionally, around 50 to 60% of surveyed students claimed theirs contributed to the development of antibiotic resistance, whenever they take an antibiotic.

The results of all attitude-related statements are mentioned in Appendix 4.

### The Practice of the Respondents Toward Antibiotics

The study showed many defects in the appropriate practices regarding antibiotic use among students in the four medical specialties, including ABSM, keeping the leftovers antibiotics, and social sharing of antibiotics.

**Fig. 1:**
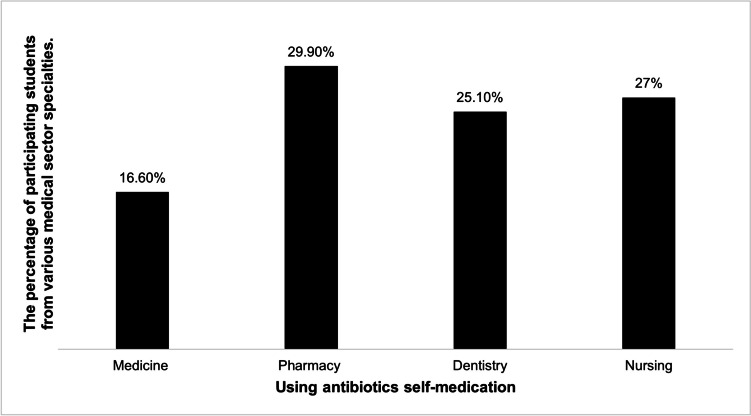
The frequency of antibiotics self-medication (ABSM) among participating students from various medical sector specialties (expressed as a percentage)

The current study revealed that PS had the highest rate in ABSM, with approximately 30% of students reporting the practice. NS followed closely, with 27% of students admitting to ABSM, while 25% of DS reported the same. On the other hand, MS had the lowest rate in ABSM, with only 16.6% of students engaging in the practice, as depicted in Fig. 1.

**Fig. 2:**
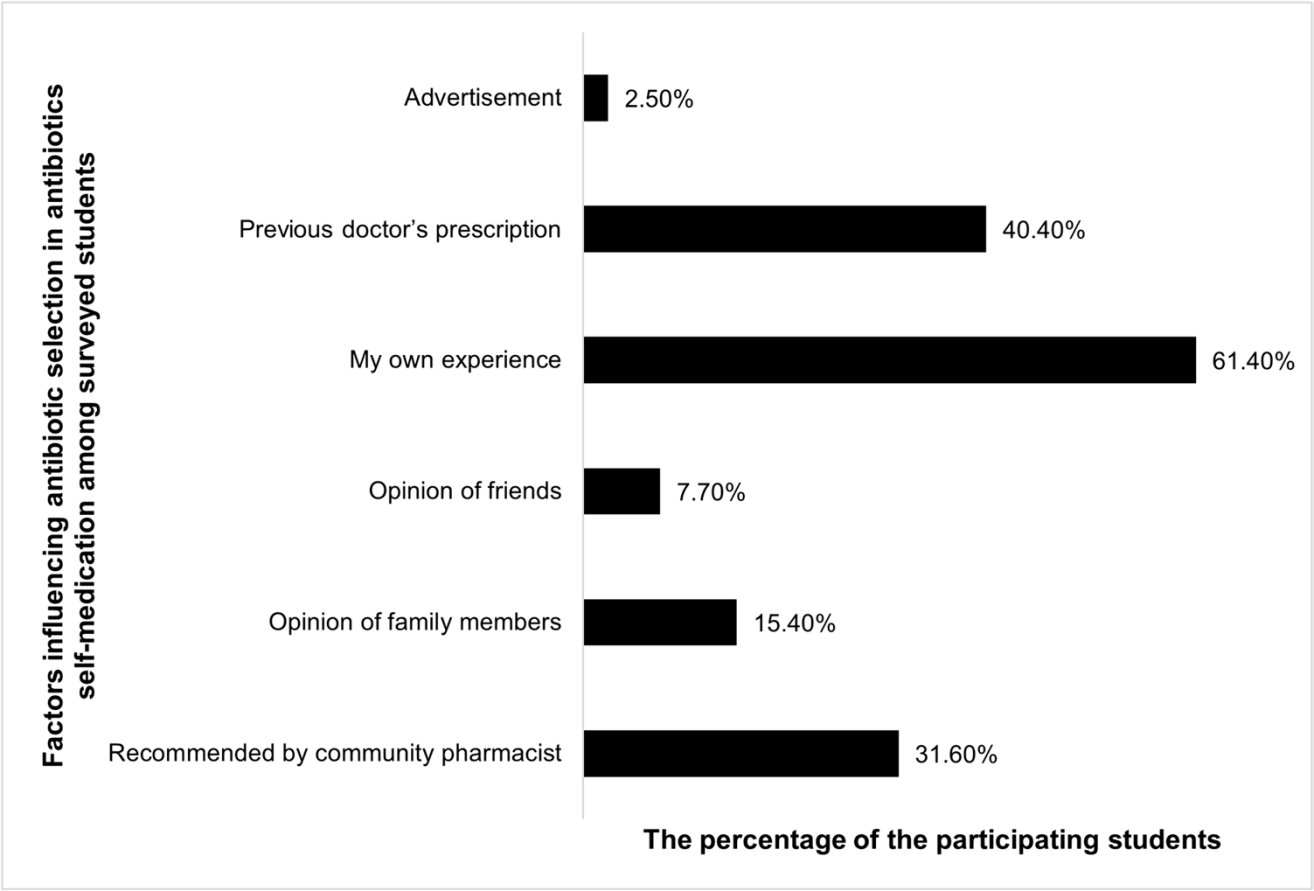
Factors influencing antibiotic selection in antibiotics self-medication (ABSM) among surveyed students (represented as percentages)

Furthermore, around 60% of surveyed students based on their antibiotic selection in ABSM on their own experience, while 40% relied on previous prescriptions, as shown in Fig. 2.

Regarding the inappropriate antibiotic practices among medical sector students, including keeping and socially sharing leftover antibiotics, the study found that around half of MS, NS, and DS stated that they kept the remaining antibiotics for future treatment. This was followed by approximately 40% of PS who reported the same practice. Approximately one-third of surveyed students reported recommending leftover antibiotics to their relatives if they got sick, indicating a practice known as social sharing, as shown in Table 4.

**Table 4:** The practice of the surveyed students about antibiotics (presented as response frequencies and percentages)

Questions of practices towards antibiotic use and its resistance (response)	Total (*N* = 1250)	MS (*N* = 217)	PS (*N* = 388)	DS (*N* = 291)	NS (*N* = 354)	*P*
Do you consult a doctor before starting antibiotics? (Yes)	871 (69.7)	175 (80.6)	245 (63.1)	201 (69.1)	250 (70.6)	< 0.001^a^
Pairwise comparisons (*P*)	*P*_MP_ < 0.001^a^	*P*_MD_ = 0.011^a^	*P*_MN_ = 0.021^a^	*P*_PD_ = 0.274	*P*_PN_ < 0.013^a^	*P*_DN_ = 0.160
Do you save the remaining antibiotics for the next time you get sick? (No)	641 (51.3)	96 (44.2)	230 (61.1)	146 (50.2)	162 (45.8)	< 0.001^a^
Pairwise comparisons (*P*)	*P*_MP_ < 0.001^a^	*P*_MD_ = 0.091	*P*_MN_ = 0.502	*P*_PD_ = 0.006^a^	*P*_PN_ < 0.001^a^	*P*_DN_ = 0.533
Do you give the leftover antibiotics to your friend/family if they get sick? (No)	811 (64.9)	138 (63.6)	275 (70.9)	193 (66.3)	205 (57.9)	0.006^a^
Pairwise comparisons (*P*)	*P*_MP_ = 0.178	*P*_MD_ = 0.465	*P*_MN_ = 0.177	*P*_PD_ = 0.200	*P*_PN_ < 0.001^a^	*P*_DN_ = 0.039
If you experience side effects of antibiotics: Do you stop taking the antibiotic without consulting a doctor or pharmacist? (Yes)	385 (30.8)	67 (30.9)	97 (25.0)	86 (29.6)	35 (38.1)	< 0.001^a^
Pairwise comparisons (*P*)	*P*_MP_ < 0.043^a^	*P*_MD_ = 0.233	*P*_MN_ < 0.001^a^	*P*_PD_ = 0.015^a^	*P*_PN_ < 0.001^a^	*P*_DN_ < 0.001^a^
If you took the wrong antibiotics: Do you visit the doctor immediately? (Yes)	808 (64.6)	126 (58.1)	252 (64.9)	177 (60.8)	253 (71.5)	0.013^a^
Pairwise comparisons (*P*)	*P*_MP_ = 0.130	*P*_MD_ = 0.374	*P*_MN_ = 0.001^a^	*P*_PD_ = 0.467	*P*_PN_ = 0.148	*P*_DN_ = 0.017^a^

Comparisons between groups and pairwise comparisons were used with the Chi-square Fisher exact test (the *p*-value on the right column of the table)

*Abbreviation: MS*, medicine students; *PS*, pharmacy students; *DS*, dentistry students; *NS*, nursing students; *N*, number of students; *P*, *p*-value at the level of significance < 0.05; *P*_*MP*_, *p*-value for comparing medicine and pharmacy; *P*_*MD*_, *p*-value at level of significance < 0.05 for comparing medicine and dentistry; *P*_*MN*_, *p*-value for comparing medicine and nursing; *P*_*PD*_, *p*-value for comparing pharmacy and dentistry; *P*_*PN*_, *p*-value for comparing pharmacy and nursing; *P*_*DN*_, *p*-value for comparing dentistry and nursing

^a^Statistically significant

Source of Knowledge and Topics Taught During the Undergraduate Studies About Antibiotics

Furthermore, the study revealed that a significant majority of NS, exceeding 70%, reported consulting their doctors in the event of taking the wrong antibiotics. In contrast, only 38% of NS sought to consult a doctor or pharmacist when experiencing side effects from antibiotics. Conversely, about one-third of MS, PS, and DS acknowledged refraining from consulting their doctor or pharmacist when they took the wrong antibiotics or experienced side effects of antibiotics, as shown in Table 4. Detailed results of all practice-related questions can be found in Appendix 5.

In terms of the sources of information for students about antibiotics, the study found that the majority, approximately 83.2%, relied on university courses. Websites followed as the second most common source of information, with 33.5% of surveyed students reporting its use. Conversely, only 7.4% of students reported utilizing national infectious disease guidelines, and 12% reported using international infectious disease guidelines as sources of information.

Furthermore, the study revealed that over 90% of students did not receive any type of training about antibiotics. Approximately one-half of the respondents expressed a desire for more education about antibiotics.

A comprehensive overview of the results of all questions in this section can be found in Appendix 6.

## Discussion

This descriptive cross-sectional study explored KAP levels among students with healthcare backgrounds toward antibiotic use and antibiotic resistance. All students were selected in the same academic year (final year) to achieve better results and avoid bias. In this way, the data of this study allowed us to assess and compare the KAP of antibiotics among students in various specialties in the medical sector to guide us to tackle the variances in their KAP levels and facilitate the narrowing of these variances. The findings in the study revealed some differences in basic knowledge, attitudes, and practices about antibiotics among the four specialties students.

The current study findings identified a wrong belief among the four medical specialties students regarding the efficacy of antibiotics in sore throats. This aligns with previous KAP survey studies on antibiotic use and resistance, which reported that 40 to 60% of respondents incorrectly believed antibiotics are effective for sore throats [15, 22, 27]. This misconception is concerning because using antibiotics for minor ailments like sore throats contributes to antibiotic resistance [28].

Using broad-spectrum antibiotics more than necessary was the most important factor contributing to resistance [9]. This survey found that most NS had the misconception that using a broad-spectrum antibiotic instead of treating the infection using a narrow-spectrum antibiotic is the best approach. However, almost all MS (over 85%) followed by 70% of DS and PS did not hold this misconception. This highlights the need for targeted education on the appropriate selection of antibiotics based on the type of infection among NS.

The findings of this study also revealed a lack of knowledge regarding the specific indications and mechanisms of action of antibiotics among NS. Many NS believed that antibiotics could be used as antipyretics or anti-inflammatory medications, indicating a misunderstanding of the appropriate use of antibiotics. This erroneous concept of considering antibiotics as antipyretics or anti-inflammatory medications demonstrates that there is a belief among NS that antibiotics can be widely used in the treatment of infectious and non-infectious diseases.

The present study identified that NS exhibited the lowest level of knowledge regarding antibiotics and antibiotic resistance, potentially due to inadequate education on these crucial subjects. Evaluating students’ knowledge levels is crucial for assessing their educational development and attainment. This highlights the critical need to integrate targeted and comprehensive educational interventions on antibiotics into nursing curricula. These interventions are essential for effectively bridging existing knowledge gaps and equipping nursing students to effectively address the challenges posed by antibiotic resistance in healthcare settings.

Notably, a significant proportion of DS showed a lack of awareness regarding the appropriate antibiotics for MRSA infections, with only a third demonstrating knowledge in this area. Furthermore, over half of MS and over a third of DS lacked knowledge regarding the safety of amoxicillin during pregnancy and breastfeeding. Additionally, a considerable number of DS and MS believed that antibiotics were used for treating sore throats, regardless of whether the infection was bacterial or viral. These knowledge gaps are concerning, as they signify their poor ability to differentiate between different types of infections. Since DS and MS are the future prescribers of antibiotics, the results highlight the need for more education for students in medicine and dentistry fields regarding the appropriate selection of antibiotics based on the type of infection. It is essential to equip future prescribers with the necessary knowledge and skills to make informed decisions regarding antibiotic prescriptions, ultimately reducing the misuse and overuse of these medications.

Poor infection control and excessive of antibiotics in animal farms have been identified as significant contributors to the local and global spread of antibiotic resistance [29]. However, approximately half of the surveyed students in the four specialties lacked knowledge about the impact of antibiotic misuse on animal food, as revealed in the current study. These results align with those of a previous study conducted among participating students in various medical sector specialties, highlighting the need for targeted educational interventions [30]. Thus, there is a pressing need to educate students in healthcare-related fields about the impact of antibiotic misuse in animal agriculture and its potential contribution to antibiotic resistance.

Although knowledge is expected to shape practices and attitudes about certain behaviors [31], this study did not find a significant influence of knowledge on students’ attitudes and practices towards antibiotics and antibiotic resistance. For example, although approximately three-quarters of MS, PS, and NS were able to differentiate between antibiotics and antipyretics, about two-fifths of MS and PS and one-half of DS admitted using antibiotics as antipyretics to expedite recovery from fever. These results align with another study that reported a considerable portion of the participating students using antibiotics for fever management [30]. However, treating fever with antibacterial agents without conducting microbiological investigations can misguide future HCPs in terms of rational antibiotic use and put them at risk of acquiring infections caused by antibiotic-resistant bacteria. Our study also revealed that students predominantly relied on personal experience rather than local or international guidelines when selecting antibiotics. Therefore, while tailored educational interventions can address knowledge gaps among surveyed students, they alone may not be sufficient to effectively improve antibiotic attitudes and practices. Integrating evidence-based practice training into interprofessional sessions could potentially enhance students’ attitudes and practices more effectively.

Moreover, in this study, only approximately half of the surveyed students recognized their contribution to the development of antibiotic resistance every time they used antibiotics, indicating the need for cultivating a sense of responsibility among medical sector students to combat the antibiotic resistance crisis for the benefit of patients and society.

The current study found that approximately half of NS and one-third of students in other medical specialties preferred newer and more expensive antibiotics, likely due to their intention to achieve optimal clinical outcomes with potent medications [32]. The results of this study also highlight that a small number of surveyed students in four specialties used local and international infectious disease guidelines as a source of information when selecting antibiotics. However, it is worth noting that many studies suggest that following appropriate antibiotic guidelines can result in a faster, more efficient path to judicious antibiotic usage [33–35]. Therefore, there is an urgent need to establish appropriate training on how to use relevant infectious disease guidelines for improving antibiotic selection among medical sector students.

ABSM is a prevalent practice observed worldwide, where individuals, without a prescription, diagnose themselves and procure antibiotics from pharmacies. This practice has been strongly linked to the rise of antibiotic-resistant bacteria. A noteworthy finding in this study is that rates of ABSM were not associated with the level of knowledge among medical sector students about antibiotics. Despite the high levels of antibiotic knowledge demonstrated by MS, PS, and DS, ABSM rates were not consistently associated with their knowledge levels. Notably, PS and DS, despite their high antibiotic knowledge level, displayed elevated ABSM rates. Conversely, NS, who had the lowest knowledge scores, exhibited a higher rate of ABSM. This may be attributed to the ease of purchasing antibiotics from pharmacies without a prescription, as community pharmacies in many developing countries do not comply with regulatory laws prohibiting over-the-counter (OTC) antibiotics dispensing [36]. This study’s findings emphasize the need for more effective, and stricter regulations to prevent the sale of antibiotics without a prescription.

The study findings revealed that medical sector students engage in inappropriate practices concerning antibiotics that may exacerbate antibiotic resistance. These practices include keeping leftover antibiotics for future use and social sharing antibiotics. This is consistent with a previous local study that showed a lack of appropriate practices among university students in preventing antibiotic resistance [27].

The present study revealed that almost all surveyed students had not received any formal training on antibiotics, which may explain their improper attitudes and practices. This observation aligns with prior research that indicates significant gaps in practical training on antibiotic use among final-year undergraduate medical-sector students [37]. Therefore, establishing adequate formal training on rational antibiotics is a crucial step in promoting proper antibiotic attitudes and practices among medical sector students. This training, provided by universities or professional organizations, should include interactive workshops, comprehensive training programs covering both theoretical knowledge and practical skills in antibiotic therapy, clinical rotations providing healthcare experience, continuing education post-graduation programs to keep updated on research and guidelines, simulation-based learning, effective antibiotic stewardship reinforcement, training in implementing infectious disease guidelines, participation in journal clubs, and accessing online extra-curricular courses. These efforts are essential to equip future healthcare professionals with the necessary skills to use antibiotics effectively and combat resistance.

Moreover, around half of the surveyed students expressed a desire for more education about antibiotics which is in agreement with the Minen study in a KAP survey conducted among participating students in America in 2010 which reported that more than three-fourths of students preferred more antibiotic education [38]. Similarly, a KAP survey conducted in 2022 among medical students in Dominica, West Indies, reported that 94% of the students desired additional education on antibiotics [39]. These findings underscore a critical gap in current educational approaches and emphasize the urgent need to enhance antibiotic education within medical training programs.

In conclusion, the study findings highlight the need for comprehensive and effective educational programs for medical sector students. These programs should not only focus on providing basic knowledge about antibiotics but also emphasize the crucial role of healthcare professionals in promoting rational antibiotic use and combating antibiotic resistance. It is imperative to establish formal training and ensure continuous education on the importance of judicious antibiotic use.

Additionally, the Ministry of Health and Population (MOHP) in Egypt could play a critical role in addressing the issue of antibiotic resistance by organizing regular workshops or seminars as part of healthcare providers’ continuing professional development (CPD) to educate them on proper antibiotic use. These efforts will contribute significantly to promoting the rational use of antibiotics and safeguarding public health for the future, highlighting the vital role of collaborative and sustained efforts from all stakeholders, including policymakers, HCPs, and the public towards mitigating the risks of antibiotic resistance.

Furthermore, the present study suggests that future studies should focus on designing tailored educational interventions that address misconceptions among medical sector specialties students regarding antibiotics’ prudent use, ultimately minimizing the emergence of antibiotic resistance. The study also recommends further research to investigate the impact of evidence-based practice training in shaping the students’ attitudes and practices regarding antibiotics.

## Limitation of Study

The study was conducted among medical students in Cairo, Egypt, limiting its generalizability to other regions across the country. It used a self-administered questionnaire which can introduce subjective responses. Being cross-sectional, the study cannot establish causal associations between variables.

## Conclusion

The present study identified misconceptions in basic knowledge regarding antibiotics and antibiotic resistance among students in four medical specialties, emphasizing the critical need for tailored educational interventions. While these interventions can address knowledge gaps, they alone are insufficient to improve antibiotic attitudes and practices among students. Therefore, additional training activities, such as workshops, clinical rotations, continuing education, and guidelines training, are necessary. The study also emphasizes the importance of implementing stricter regulations, including pharmacy policies, to prevent non-prescription antibiotic sales and encourages interdisciplinary collaboration to effectively combat antibiotic resistance. Implementing these measures could significantly improve the knowledge, attitudes, and practices of future HCPs, thereby playing a vital role in addressing antibiotic resistance.

## Supplementary Information

Below is the link to the electronic supplementary material.

Supplementary file1 (PDF 297 KB)

Supplementary file2 (PDF 16 KB)

Supplementary file3 (PDF 239 KB)

Supplementary file4 (PDF 144 KB)

Supplementary file5 (PDF 388 KB)

Supplementary file6 (PDF 474 KB)

## Data Availability

Data is available upon proper request to the corresponding author.

## Declarations

We confirm that neither the manuscript nor any parts of its content are currently under consideration or published in another journal.
