# Supplementary material for: Knowledge, Attitude, and Practice Towards Antibiotics Use Among Medical Sector Final-Year Students in Egypt
Source: Med Sci Educ. 2024 Aug 2;34(6):1369–79. doi: 10.1007/s40670-024-02117-6 (PMC11698705; doi:10.1007/s40670-024-02117-6)
Supplement: Supplementary file 1 — Supplementary file1 (PDF 297 KB) [file 40670_2024_2117_MOESM1_ESM.pdf]

Article title: Knowledge, Attitude, and Practice Towards Antibiotics Use Among Medical Sector Final-Year Students in Egypt.

Journal name: Medical Science Educator

Author name: Nourhan M. Emera

Email address: Nourhan.mo.emera@pharma.cu.edu.eg

Appendix 1The final version of the KAP study questionnaire

Knowledge, Attitude, and Practice Towards Antibiotics Use Among Medical Sector Final-Year Students in Egypt.

Date…………………. **Serial Number…………………..**

Ending time…………………. **Starting time……………………….**

Dear student,

This survey is a part of a study conducted to assess medical student's knowledge and attitude toward antibiotic use, at the Faculty of Pharmacy, Cairo University. We would be grateful if you participated in this study.

Part 1: Basic demographic data:

Gender: □ Male □ Female **Age: ……………………**

University of graduation: ……………………………

□ Nursing **□ Dentistry** **□ Pharmacy** **□ Medicine**

Part 2: Knowledge about Antibiotics (The answer should be based on your knowledge, not what should be or the ideal situation):

	Item	True	False	Do not know
K.1	Antibiotics work mainly against ***(You are requested to select False, True, or Do not know for each presented option from A-C)***			
	A. Viral infections			
	B. Bacterial infections			
	C. Fungal infection.			
K.2	Antibiotics are used for ***(You are requested to select False, True, or Do not know for each presented option)***			
	A. Common cold, flu, cough, and nasal congestion			
	B. Non-febrile diarrhea in children			
	C. Sore throat			
K.3	Use of Broad-spectrum antibiotics is better than narrow-spectrum ones			
K.4	Antibiotics are obtainable without interference from a doctor at pharmacies (i.e. Antibiotics are over-the-counter drugs)			
K.5	Antibiotics are considered anti-inflammatory medications.			
K.6	Antibiotics have antipyretic effects.			
K.7	Methicillin resistant Staphylococcus aureus (MRSA) is susceptible to ***(You are requested to select False, True, or Do not know for each presented option from A to C)***			
	A. Amoxicillin			
	B. Cefotaxime			
	C. Vancomycin			
K.8	The antibiotic is considered safe to use during the first trimester of pregnancy and breastfeeding.			
	A. Levofloxacin			
	B. Amoxicillin			

	Item	True	False	Do not know
K.9	Antibiotic usage disturbs the gut flora and causes diarrhea and super-infection			
K.10	Tetracycline could be harmful to a child's teeth.			
K.11	Antibiotics might cause an allergy leading to death			
K.12	Antibiotics are advised to be given: ***(You are requested to select False, True, or Do not know for each presented option***			
	A. With plenty of water			
	B.Milk.			
K.13	Other medications can influence the effect of antibiotics.			
K.14	Antibiotics can influence the effect of other medications.			
K.15	Antibiotic abuse is a serious problem in Egypt.			
K.16	Antibiotic resistance is a phenomenon by which bacteria lose their sensitivity.			
K.17	Inappropriate use of antibiotics can lead to ***(You are requested to select False, True, or Do not know for each presented option from A to F)***			
	A. Ineffective treatment.			
	B. Increased adverse effect.			
	C. Aggravation or prolongation of illness.			
	D. Emergence of bacterial resistance.			
	E. An additional medical cost to the patient.			
	F. Other (Please specify): …………			
K.18	Antibiotic resistance is due to***(You are requested to select False, True, or Do not know for each presented option from A to F)***			
	A. Using antibiotics when they are not necessary.			
	B. Not completing the full course of antibiotics.			
	C. Using antibiotics without a physician's prescription (Self-medication).			

	D. Using broad-spectrum antibiotics more than necessary spectrum.			
	E. Excessive antibiotic use in animal food (cattle, sheep, poultry,………..).			
	F. Others (Please specify):………………………………			

Part 3: Attitude regarding antibiotic use and resistance: *only one answer is required

	Item	Yes	No	Unsure
A.1	When you get a fever, antibiotics help you to get better faster.			
A.2	When you have a cold, you should take antibiotics to prevent getting a more serious illness.			
A.3	You select more expensive and newer antibiotics to provide more effective action and fewer side effects			
A.4	It is acceptable to skip one or two doses of antibiotics as long the whole course will be continued			
A.5	You can reduce the dose of antibiotics without consulting your doctor			
	**Item**	**Yes**	**No**	**Unsure**
A.6	You can take antibiotics at different times each day as long as the daily doses are taken.			
A.7	You stop antibiotic use without consulting your doctor			
A.8	You request an antibiotic prescription from your doctor even if the doctor has advised against it			
A.9	You follow the doctor's instructions when prescribed antibiotics			
A.10	You believe that antibiotics should be prescribed only after culture and sensitivity report			
A.11	You ask your physician for an antibiotic allergy test			
A.12	You contribute to the development of antibiotic resistance, whenever you take an antibiotic			
A.13	Antibiotic resistance can affect you & your family's health			

Part 4: Practice towards antibiotics:

P.1. What illness/symptoms have you had in the last six months? ***((More than one option can be selected if applicable))***

☐ Common cold ☐ Runny nose ☐ Cough ☐ Sore throat

☐ Fever ☐ Abdominal pain ☐ Headache ☐ Diarrhea

☐ Others (Specify): **……………………………………………………………………….**

P.2. What have you done for an illness you have experienced in the last six months?

☐ Nothing ☐Seen a doctor ☐ Self-medicated

straight to P.4 If seen a doctor or nothing, go

P.3.1. If you were self-medicated, did you use antibiotics?

☐ Yes ☐ No

If No, go straight to P.4.

P.3.2. Please write down the names of antibiotics you have taken for self-medication:

……
………………………………………………………………………….

P.3.3. Your selection of antibiotics was based on? ***((More than one option can be selected if applicable))***

☐Opinion of family members ☐ Recommended by the community pharmacist

☐ My own experience ☐ Opinion of friends

☐ Advertisement ☐ Previous doctor's prescription

☐ **Others………………………………………**

P.3.4 What did/do you consider when selecting antibiotics? ***((More than one option can be selected if applicable))***

☐ Price of antibiotics ☐ Brand of antibiotics ☐ Type of antibiotics

☐ Indications for use ☐ Adverse reactions ☐ **Others…………….**

P.3.5. What was(were) your reason(s) for self-medication with antibiotics? ***((More than one option can be selected if applicable))***

☐ Lack of trust in prescribing doctor ☐ Convenience ☐ Cost saving

☐ **Others** (specify**):……………………………………………………………….**

Item		Yes	No	Unsure
P.4	Do you consult a doctor before starting antibiotics?			
P.5	*The Doctor prescribes a course of antibiotics for you. After taking 2–3 doses you start feeling better* ***(You are requested to select False, True, or Do not know for each presented option from A to C)***			
	A. Do you stop taking the further treatment?			
	B. Do you complete the full course of treatment?			
	C. Do you save the remaining antibiotics for the next time you get sick?			
	D. Do you discard the remaining antibiotics?			
	E. Do you give the leftover antibiotics to your friends/family if they get sick?			
P.6	*If you experience side effects of antibiotics:*<br><br>Do you stop taking the antibiotic without consulting a doctor or pharmacist?			
P.7	*If you took the wrong antibiotics,*<br><br>Do you visit the doctor immediately?			
P.8	You read the instructions in the package insert & check the expiry date before taking antibiotics.			

Part 5: Your sources of information about antibiotics and the topics taught in the medical curriculum.

S.1. Your source of information about antibiotic selection is/are? **((More than one option can be selected if applicable))**

☐ University course

☐ PharmaceuticalCompaniess

☐**International infectious disease guidelines**

☐ Website

☐ **National infectious disease guidelines**

☐ **(**Others (Specify)……………………….

S.2. Your source of information regarding the directions of the use of antibiotics in your illness: **((More than one option can be selected if applicable))**

□ Relatives and Friends □ Pharmacist □ Physician

□ Previous experience □ Drug information leaflet □ Others (Specify): ....

T.1.1 In particular, have the problem of antibiotic resistance been discussed during any of your undergraduate courses?

□ Yes □ No

T.1.2 If yes, in which courses/specialty?
....

T.2.1. Have you ever heard of antibiotic resistance outside your undergraduate courses?

□ Yes □ No

T.2.2. If yes, where have you heard about it from? **((More than one option can be selected if applicable))**

□ Web □ Newspaper □ General Practitioner □ Television

□ Other ....

T.3 Which of the following topic(s) was(were) **not** being taught in your undergraduate curricula? **(More than one option can be selected if applicable))**

□ Understanding the mechanism of action of antibiotics, & chemical structures, and spectrum of activity of antibiotics

□ Interpretive basic microbiological investigations (e.g. Blood cultures, antibiotic susceptibility reporting).

□ Adverse drug, contra-indication, and allergy reactions of antibiotics.

□ Pharmacokinetics of antibiotics and Therapeutic drug monitoring.

□ Differentiation between bacterial and viral infection.

□ Negative consequences of antibiotic use (bacterial resistance, cost, etc)

□ Pharmaceutical calculation, dosing of antibiotics & route administration of antibiotics.

□ Understanding social issues in the use of antibiotics □ Selective toxicity of antibiotics

□ Infection prevention and control □ Rational use of antibiotics in special patients

□ Therapeutic drug monitoring □ Classification according to the spectrum of activity

□ All the above be covered.

T.4.1 Have you received any type of training about antibiotics?

□ Yes □ No

T.4.2 If yes, Specify…………………………………………………….

T.5.1 Do you think that you need more education about antibiotics?

□Yes □ No □Unsure

T.5.2 If yes, what topics do you think should be included in medical curricula about antibiotics? ………………………………………………………………………………………………………

………
…………………………………………………………………………

………
………
………
………...
