# Supplementary material for: Knowledge, Attitude, and Practice Towards Antibiotics Use Among Medical Sector Final-Year Students in Egypt
Source: Med Sci Educ. 2024 Aug 2;34(6):1369–79. doi: 10.1007/s40670-024-02117-6 (PMC11698705; doi:10.1007/s40670-024-02117-6)
Supplement: Supplementary file 2 — Supplementary file2 (PDF 16 KB) [file 40670_2024_2117_MOESM2_ESM.pdf]

Article title: Knowledge, Attitude, and Practice Towards Antibiotics Use Among Medical Sector Final-Year Students in Egypt.

Journal name: Medical Science Educator

Author name: Nourhan M. Emera

Email address: Nourhan.mo.emera@pharma.cu.edu.eg

Appendix 2 List of enrolled medical sector and health care discipline faculties.

**A. Dentistry specialty**	**B. Medicine specialty**	**C. Nursing specialty**	**D. Pharmacy specialty**
1. The faculty of dentistry- ASU	7. The faculty of medicine- ASU	11. The faculty of nursing- ASU	17. The faculty of pharmacy- ASU
2. The faculty of dentistry-O6U	8. The faculty of medicine- O6U	12. The faculty of nursing- O6U	18. The faculty of pharmacy- O6U
3. The faculty of dentistry-MUST	9. The faculty of medicine- AZU	13. The faculty of nursing- AZU	19. The faculty of pharmacy- AZU
4. The faculty of dentistry-CU	10. The faculty of medicine- MUST	14. The faculty of nursing- HU	20. The faculty of pharmacy- HU
5. The faculty of dentistry-FUE		15. The faculty of nursing- CU	21. The faculty of pharmacy- CU
		16. The faculty of nursing- MTI	22. The faculty of pharmacy- MTI
			23. The faculty of pharmacy- MUST
			24. The faculty of pharmacy- FUE
			25. The faculty of pharmacy- BUE

ASU: Ain Shams University, O6U: October 6 University, MUST: Misr University for Science and Technology, CU: Cairo University, FUE: Future University in Egypt, AZU: al-Azhar University, HU: Helwan University, MTI: modern university for Technology and information, BUE: British University in Egypt.
