# Supplementary material for: Knowledge, Attitude, and Practice Towards Antibiotics Use Among Medical Sector Final-Year Students in Egypt
Source: Med Sci Educ. 2024 Aug 2;34(6):1369–79. doi: 10.1007/s40670-024-02117-6 (PMC11698705; doi:10.1007/s40670-024-02117-6)
Supplement: Supplementary file 3 — Supplementary file3 (PDF 239 KB) [file 40670_2024_2117_MOESM3_ESM.pdf]

Article title: Knowledge, Attitude, and Practice Towards Antibiotics Use Among Medical Sector Final-Year Students in Egypt.

Journal name: Medical Science Educator

Author name: Nourhan M. Emera

Email address: Nourhan.mo.emera@pharma.cu.edu.eg

Appendix 3. Details on all knowledge-related questions of the respondents' students in the four medical specialties.

Table 2. Students' responses (expressed as frequency (%) of correct answers) to questions related to knowledge about antibiotic use and antibiotic resistance.

Knowledge questions	Total (N = 1250)	MS (N= 217)	PS (N= 388)	DS (N = 291)	NS (N = 354)	P
K.1.Antibiotics work mainly against						
A. Viral infections	1180(94.4)	217 (100.0)	380(97.9)	281 (96.6)	302(85.3)	< 0.001 [a]
Pairwise comparisons (P)	P_{MP} = 0.056	P_{MD} = 0.006 [a]	P_{MN} < 0.001 [a]	P_{PD} =0.270	P_{PN} < 0.001 [a]	P_{DN} < 0.001 [a]
B. Bacterial infections	1239 (99.1)	217(100.0)	388(100.0)	287(98.6)	347(98)	0.004 [a]
Pairwise comparisons (P)	P_{MP} = …..	P_{MD} 0.139	P_{MN} < 0.048 [a]	P_{PD} < 0.033 [a]	P_{PN} < 0.005 [a]	P_{DN} =0.762

C. Fungal infections	1093 (87.4)	212 (97.7)	328(84.5)	262 (90.0)	291 (82.2)	< 0.001 [a]
Pairwise comparisons (P)	P_{MP}<br>< 0.001 [a]	P_{MD}<br>= 0.001 [a]	P_{MN}<br>< 0.001 [a]	P_{PD}<br>=0.036 [a]	P_{PN}<br>=0.393	P_{DN}<br>< 0.005 [a]
K.2. Antibiotics are used for:						
A. Common cold, flu, cough and nasal congestion	1005 (80.4)	206 (94.9)	350 (90.2)	215 (73.9)	234 (66.1)	< 0.001 [a]
Pairwise comparisons (P)	P_{MP}<br>= 0.041 [a]	P_{MD}<br>< 0.001 [a]	P_{MN}<br>< 0.001 [a]	P_{PD}<br>< 0.001 [a]	P_{PN}<br>< 0.001 [a]	P_{DN}<br>= 0.033 [a]
B. Non-febrile diarrhea in children	954 (76.3)	197 (90.8)	286 (73.7)	224 (77.0)	247 (69.8)	<0.001 [a]
Pairwise comparisons (P)	P_{MP}<br>< 0.001 [a]	P_{MD}<br>< 0.001 [a]	P_{MN}<br>< 0.001 [a]	P_{PD}<br>= 0.330	P_{PN}<br>= 0.234	P_{DN}<br>< 0.040 [a]
C. Antibiotics are used for sore throat	508 (40.6)	134 (61.8)	129 (33.2)	142 (48.8)	103 (29.1)	<0.001 [a]
Pairwise comparisons (P)	P_{MP}<br>< 0.001 [a]	P_{MD}<br>= 0.004 [a]	P_{MN}<br>< 0.001 [a]	P_{PD}<br>< 0.001 [a]	P_{PN}<br>= 0.223	P_{DN}<br>< 0.001 [a]
K.3. The use of broad-spectrum antibiotics is better than narrow-spectrum ones.	812 (65.0)	185 (85.3)	267 (68.8)	199 (68.4)	161 (45.5)	<0.001 [a]

Pairwise comparisons (P)	P_{MP}	P_{MD}	P_{MN}	P_{PD}	P_{PN}	P_{DN}
	< 0.001 [a]	= 0.001 [a]	< 0.001 [a]	= 0.905	< 0.001 [a]	< 0.001 [a]
K.4. Antibiotics are obtainable without interference of a doctor at pharmacies (i.e. Antibiotics are over-the-counter drugs).	1037 (83.0)	183 (84.3)	345 (88.9)	239 (82.1)	270 (76.3)	<0.001 [a]
Pairwise comparisons (P)	P_{MP}	P_{MD}	P_{MN}	P_{PD}	P_{PN}	P_{DN}
	=0.105	= 0.513	= 0.021 [a]	= 0.012	< 0.001 [a]	= 0.069
K.5. Antibiotics are considered anti-inflammatory medications.	972 (77.8)	194 (89.4)	341 (87.9)	233 (80.1)	204 (57.6)	<0.001 [a]
Pairwise comparisons (P)	P_{MP}	P_{MD}	P_{MN}	P_{PD}	P_{PN}	P_{DN}
	= 0.577	= 0.004 [a]	< 0.012 [a]	< 0.005 [a]	< 0.001 [a]	< 0.001 [a]
K.6. Antibiotics have antipyretic effects.	825 (66.0)	159 (73.3)	276 (71.1)	202 (69.4)	188 (53.1)	<0.001 [a]
Pairwise comparisons (P)	P_{MP}	P_{MD}	P_{MN}	P_{PD}	P_{PN}	P_{DN}
	= 0.575	= 0.349	< 0.001 [a]	= 0.627	< 0.001 [a]	< 0.001 [a]
K.7.MRSA is susceptible to:						
A. Amoxicillin	669 (53.5)	146 (67.3)	293 (75.5)	127 (43.6)	103 (29.1)	< 0.001 [a]
Pairwise comparisons (P)	P_{MP}	P_{MD}	P_{MN}	P_{PD}	P_{PN}	P_{DN}
	= 0.029 [a]	< 0.001 [a]	< 0.001 [a]	< 0.001 [a]	< 0.001 [a]	< 0.001 [a]
B. Cefataxime	809 (64.7)	159 (73.3)	290 (74.7)	183 (62.9)	177 (50.0)	< 0.001 [a]
Pairwise comparisons (P)	P_{MP}	P_{MD}	P_{MN}	P_{PD}	P_{PN}	P_{DN}
	= 0.692	= 0.014 [a]	< 0.001 [a]	< 0.001 [a]	< 0.001 [a]	= 0.001 [a]

C. Vancomycin	733 (58.6)	160 (73.7)	317 (81.7)	119 (40.9)	137 (38.7)	<0.001 [a]
Pairwise comparisons (P)	P_{MP} = 0.021 [a]	P_{MD} < 0.001 [a]	P_{MN} < 0.001 [a]	P_{PD} < 0.001 [a]	P_{PN} < 0.001 [a]	P_{DN} = 0.571
K.8. The antibiotic which is considered safe to use during the first trimester of pregnancy and breastfeeding.						
A. Levofloxacillin	640 (51.2)	106 (48.8)	237 (61.1)	167 (57.4)	130 (36.7)	<0.001 [a]
Pairwise comparisons (P)	P_{MP} = 0.004 [a]	P_{MD} =0.056	P_{MN} = 0.004 [a]	P_{PD} = 0.332	P_{PN} < 0.001 [a]	P_{DN} < 0.001 [a]
B. Amoxicillin	645 (51.6)	106 (48.8)	242 (62.4)	174 (59.8)	123 (34.7)	<0.001 [a]
Pairwise comparisons (P)	P_{MP} < 0.001 [a]	P_{MD} = 0.014 [a]	P_{MN} < 0.001 [a]	P_{PD} = 0.495	P_{PN} < 0.001	P_{DN} < 0.001 [a]
K.9. Antibiotic usage disturbs the gut flora and causes diarrhea and super-infection	952 (76.2)	197 (90.8)	339 (87.4)	223 (76.6)	193 (54.5)	<0.001 [a]
Pairwise comparisons (P)	P_{MP} = 0.205	P_{MD} = 0.001 [a]	P_{MN} < 0.001 [a]	P_{PD} < 0.001 [a]	P_{PN} < 0.001 [a]	P_{DN} < 0.001 [a]
K.10. Tetracycline could be harmful to a child's teeth.	975 (78.0)	180 (82.9)	367 (94.6)	280 (96.2)	148 (41.8)	<0.001 [a]
Pairwise comparisons (P)	P_{MP} < 0.001[a]	P_{MD} = 0.004[a]	P_{MN} < 0.001[a]	P_{PD} = 0.321	P_{PN} < 0.001[a]	P_{DN} < 0.001 [a]

K.11.Antibiotics might cause an allergy leading to death	1077 (86.2)	190 (87.6)	353 (91.0)	255 (87.6)	279 (78.8)	<0.001 [a]
Pairwise comparisons (P)	P_{MP} =0.183	P_{MD} = 0.981	P_{MN} = 0. 008[a]	P_{PD} =0.158	P_{PN} < 0.001[a]	P_{DN} = 0.003 [a]
K.12. Antibiotics are advised to be given:						
A. With plenty of water	1106 (88.5)	168 (77.4)	360 (92.8)	265 (91.1)	313 (88.4)	<0.001 [a]
Pairwise comparisons (P)	P_{MP} < 0.001[a]	P_{MD} < 0.001[a]	P_{MN} < 0.001[a]	P_{PD} = 0.413	P_{PN} = 0.041[a]	P_{DN} = 0.273
B. Milk	1059 (84.7)	171 (78.8)	354 (91.2)	242 (83.2)	292 (82.5)	<0.001 [a]
Pairwise comparisons (P)	P_{MP} < 0.001 [a]	P_{MD} = 0.213	P_{MN} = 0.275	P_{PD} < 0.091[a]	P_{PN} < 0.001 [a]	P_{DN} = 0.821
K.13. Other medications can influence the effect of antibiotics.	1116 (89.3)	197 (90.8)	355 (91.5)	252 (86.6)	312 (88.1)	0.160
K.14. Antibiotics can influence the effect of other medications.	1093 (87.4)	187 (86.2)	345 (88.9)	253 (86.9)	308 (87.0)	0.750
K.15. Antibiotic abuse is a serious problem in Egypt.	1194 (95.5)	214 (98.6)	380 (97.9)	280 (96.2)	320 (90.4)	<0.001 [a]
Pairwise comparisons (P)	P_{MP} =0.754	P_{MD} = 0.102	P_{MN} < 0.001[a]	P_{PD} 0.179	P_{PN} < 0.001[a]	P_{DN} = 0.004[a]
K.16. Antibiotic resistance is a phenomenon by which bacteria loses its sensitivity.	1014 (81.1)	196 (90.3)	329 (84.8)	237 (81.4)	252 (71.2)	< 0.001[a]
Pairwise comparisons (P)	P_{MP} =0.054	P_{MD} = 0.005[a]	P_{MN} < 0.001[a]	P_{PD} =0.246	P_{PN} < 0.001[a]	P_{DN} = 0.002[a]

K.17. Inappropriate use of antibiotics can lead to:						
A. Ineffective treatment.	1157 (92.6)	214 (98.6)	364 (93.8)	266 (91.4)	313 (88.4)	< 0.001[a]
Pairwise comparisons (P)	P_{MP} =0.006 [a]	P_{MD} < 0.001[a]	P_{MN} < 0.001[a]	P_{PD} =0.231	P_{PN} = 0.009[a]	P_{DN} = 0.212
B. Increased adverse effect.	1047 (83.8)	188 (86.6)	328 (84.5)	235 (80.8)	296 (83.6)	0.330
C. Aggravation or prolongation of illness.	1027 (82.2)	190 (87.6)	354 (91.2)	218 (74.9)	265 (74.9)	< 0.001[a]
Pairwise comparisons (P)	P_{MP} =0.149	P_{MD} < 0.001[a]	P_{MN} < 0.001[a]	P_{PD} < 0.001[a]	P_{PN} < 0.001[a]	P_{DN} = 0.987
D. Emergence of bacterial resistance.	1155 (92.4)	211 (97.2)	376 (96.9)	268 (92.1)	300 (84.7)	< 0.001[a]
Pairwise comparisons (P)	P_{MP} =0.820	P_{MD} = 0.014[a]	P_{MN} < 0.001[a]	P_{PD} = 0.005[a]	P_{PN} < 0.001[a]	P_{DN} = 0.004[a]
E .An additional medical cost to the patient.	1126 (90.1)	202 (93.1)	369 (95.1)	253 (86.9)	302 (85.3)	< 0.001[a]
Pairwise comparisons (P)	P_{MP} =0.302	P_{MD} = 0.025[a]	P_{MN} = 0.005[a]	P_{PD} < 0.001[a]	P_{PN} < 0.001[a]	P_{DN} = 0.552
K.18. Antibiotic resistance is due to:						
A. Using antibiotics when they are not necessary.	1160 (92.8)	213 (98.2)	372 (95.9)	278 (95.5)	297 (83.9)	< 0.001[a]
Pairwise comparisons (P)	P_{MP} =0.132	P_{MD} = 0.104	P_{MN} < 0.001[a]	P_{PD} =0.827	P_{PN} < 0.001[a]	P_{DN} < 0.001[a]
B. Not completing the full course of antibiotic.	1097 (87.8)	203 (93.5)	372 (95.9)	244 (83.8)	278 (78.5)	< 0.001[a]

Pairwise comparisons (P)	P_{MP} = 0.206	P_{MD} = 0.001[a]	P_{MN} < 0.001[a]	P_{PD} < 0.001[a]	P_{PN} < 0.001[a]	P_{DN} = 0.087
C. Using antibiotics without physician prescription (Self-medication).	1134 (90.7)	209 (96.3)	371 (95.6)	264 (90.7)	290 (81.9)	< 0.001[a]
Pairwise comparisons (P)	P_{MP} = 0.680	P_{MD} = 0.014[a]	P_{MN} < 0.001[a]	P_{PD} =0.010[a]	P_{PN} < 0.001[a]	P_{DN} =0.001[a]
D. Using broad spectrum antibiotics more than necessary spectrum.	1034 (82.7)	195 (89.9)	354 (91.2)	240 (82.5)	245 (69.2)	< 0.001[a]
Pairwise comparisons (P)	P_{MP} = 0.576	P_{MD} = 0.019	P_{MN} < 0.001[a]	P_{PD} = 0.001[a]	P_{PN} < 0.001[a]	P_{DN} < 0.001[a]
E. Excessive antibiotic use in animal food (cattle, sheep, poultry)	672 (53.8)	112 (51.6)	239 (61.6)	139 (47.8)	182 (51.4)	0.002 [a]
Pairwise comparisons (P)	P_{MP} < 0.017 [a]	P_{MD} = 0.391	P_{MN} = 0.963	P_{PD} < 0.001[a]	P_{PN} = 0.005[a]	P_{DN} = 0.357

Abbreviations: MRSA = Methicillin resistant staphylococcus aureus, MS = Medicine students, PS= Pharmacy students, DS = Dentistry students, NS = Nursing students, N: Number of students.

Comparison between groups and pairwise comparisons using the Chi-square Fisher Exact test for categorical variables and Kruskal-Wallis for non-normally distributed continuous variables (the p-value on the right column of the table).

P: p-value at level of significance < 0.05, P_{MP}: p-value for comparing medicine and pharmacy, P_{MD}: p-value for comparing medicine and dentistry, P_{MN}: p-value for comparing medicine and nursing, P_{PD}: p-value for comparing pharmacy and dentistry, P_{PN}: p-value for comparing pharmacy and nursing, P_{DN}: p-value for comparing dentistry and nursing.

[a]: statistically significant.
