# Supplementary material for: Knowledge, Attitude, and Practice Towards Antibiotics Use Among Medical Sector Final-Year Students in Egypt
Source: Med Sci Educ. 2024 Aug 2;34(6):1369–79. doi: 10.1007/s40670-024-02117-6 (PMC11698705; doi:10.1007/s40670-024-02117-6)
Supplement: Supplementary file 4 — Supplementary file4 (PDF 144 KB) [file 40670_2024_2117_MOESM4_ESM.pdf]

Article title: Knowledge, Attitude, and Practice Towards Antibiotics Use Among Medical Sector Final-Year Students in Egypt.

Journal name: Medical Science Educator

Author name: Nourhan M. Emera

Email address: Nourhan.mo.emera@pharma.cu.edu.eg

Appendix 4. The results of all attitude-related statements.

The attitude of the surveyed students about antibiotics and antibiotic resistance (presented as response frequencies (%)).

Questions of attitudes towards antibiotic use and its resistance (Response)	Total (N = 1250)	MS (N = 217)	PS (N = 388)	DS (N = 291)	NS (N = 354)	P
A.1. When you get a fever, antibiotics help you to get better faster (No)	686 (54.9)	141 (65.0)	234 (60.3)	147 (50.5)	164 (46.3)	<0.001[a]
Pairwise comparisons (P)	P_{MP}	P_{MD}	P_{MN}	P_{PD}	P_{PN}	P_{DN}
	= 0.106	= 0.002[a]	< 0.001[a]	< 0.036[a]	< 0.001[a]	= 0.028[a]
A.2. When you have a cold, you should take antibiotics to prevent getting a more serious illness. (No)	974 (77.9)	176 (81.1)	331 (85.3)	243 (83.5)	224 (63.3)	<0.001[a]
Pairwise comparisons (P)	P_{MP}	P_{MD}	P_{MN}	P_{PD}	P_{PN}	P_{DN}

	= 0.398	= 0.0647	< 0.001	= 0.723	< 0.001[a]	< 0.001[a]
A.3. You select more expensive and newer antibiotics to provide more effective action and fewer side effects (No)	776 (62.1)	131 (60.4)	267 (68.8)	188 (64.6)	190 (53.7)	<0.001[a]
Pairwise comparisons (P)	P_{MP} = 0.054	P_{MD} = 0.180	P_{MN} = 0.292	P_{PD} = 0.453	P_{PN} < 0.001[a]	P_{DN} = 0.003[a]
A.4. It is acceptable to skip one or two doses of antibiotics as long the whole course will be continued (No)	946 (75.7)	164 (75.6)	323 (83.2)	234 (80.4)	225 (63.6)	<0.001[a]
Pairwise comparisons (P)	P_{MP} = 0.064	P_{MD} = 0.308	P_{MN} = 0.001[a]	P_{PD} = 0.607	P_{PN} < 0.001[a]	P_{DN} < 0.001[a]
A.5. You can reduce the dose of antibiotics without consulting your doctor (No)	1087 (87.0)	203(93.5)	347 (89.4)	266(91.4)	271(76.6)	< 0.001[a]
Pairwise comparisons (P)	P_{MP} = 0.240	P_{MD} = 0.354	P_{MN} < 0.001[a]	P_{PD} =0.344	P_{PN} < 0.001[a]	P_{DN} <0.001[a]
A.6. You can take antibiotics at different times each day as long the daily doses are taken. (No)	988 (79.1)	177(81.6)	322 (83.0)	244(84.1)	245(69.2)	< 0.001[a]
Pairwise comparisons (P)	P_{MP} = 0.901	P_{MD} = 0.747	P_{MN} = 0.004[a]	P_{PD} = 0.919	P_{PN} < 0.001[a]	P_{DN} <0.001[a]
A.7. You stop antibiotics use without consulting your doctor (No)	974 (77.9)	181(83.4)	317 (81.7)	247 (84.9)	229 (64.7)	< 0.001[a]

Pairwise comparisons (P)	P_{MP} = 0.291	P_{MD} = 0.695	P_{MN} < 0.001[a]	P_{PD} =0.429	P_{PN} < 0.001[a]	P_{DN} < 0.001[a]
A.8. You request an antibiotic prescription from your doctor even if the doctor has advised against it (No)	1017 (81.4)	191 (88.0)	315 (81.2)	257 (88.3)	254 (71.8)	< 0.001[a]
Pairwise comparisons (P)	P_{MP} = 0.010 [a]	P_{MD} = 0.320	P_{MN} < 0.001[a]	P_{PD} = 0.037[a]	P_{PN} = 0.008[a]	P_{DN} < 0.001[a]
A.9. You follow doctor's instruction when prescribed antibiotics (Yes)	1129 (90.3)	200 (92.2)	339 (87.4)	273 (93.8)	317 (89.5)	0.004[a]
Pairwise comparisons (P)	P_{MP} = 0.192	P_{MD} = 0.194	P_{MN} =0.239	P_{PD} = 0.001[a]	P_{PN} =0.167	P_{DN} = 0.018[a]
A.10. You believe that antibiotics should be prescribed only after culture and sensitivity report (Yes)	906(72.5)	145 (66.8)	296 (76.3)	216 (74.2)	249 (70.3)	0.243
A.11. You ask your physician for antibiotic allergy (Yes)	897 (71.8)	140 (64.5)	292 (75.3)	207 (71.1)	258 (72.9)	0.160
A.12. You contribute to the development of antibiotic resistance, whenever	738(59.0)	110(50.7)	228(58.8)	177(60.8)	223(63.0)	0.038[a]
Pairwise comparisons (P)	P_{MP} =0.120	P_{MD} = 0.057	P_{MN} = 0.008 [a]	P_{PD} =0.258	P_{PN} = 0.071	P_{DN} = 0.806
A.13.You contribute to the development of antibiotic resistance, whenever you take an antibiotic (Yes)	738 (59.0)	110 (50.7)	228 (58.8)	177 (60.8)	223 (63.0)	0.038[a]

Pairwise comparisons (P)	P_{MP} = 0.120	P_{MD} = 0.057	P_{MN} < 0.008[a]	P_{PD} = 0.258	P_{PN} < 0.071	P_{DN} = 0.806

Abbreviations: MS = Medicine students, PS= Pharmacy students, DS = Dentistry students, NS = Nursing students, N: Number of students.

Comparison between groups and pairwise comparisons were used with the Chi-square Fisher Exact test (the p-value on the right column of the table). P: p-value at level of significance < 0.05, P_{MP}: p-value for comparing medicine and pharmacy, P_{MD}: p-value for comparing medicine and dentistry, P_{MN}: p-value for comparing medicine and nursing, P_{PD}: p-value for comparing pharmacy and dentistry, P_{PN}: p-value for comparing pharmacy and nursing, P_{DN}: p-value for comparing dentistry and nursing.

[a]: statistically significant.
