# Supplementary material for: Knowledge, Attitude, and Practice Towards Antibiotics Use Among Medical Sector Final-Year Students in Egypt
Source: Med Sci Educ. 2024 Aug 2;34(6):1369–79. doi: 10.1007/s40670-024-02117-6 (PMC11698705; doi:10.1007/s40670-024-02117-6)
Supplement: Supplementary file 5 — Supplementary file5 (PDF 388 KB) [file 40670_2024_2117_MOESM5_ESM.pdf]

**Article title:** Knowledge, Attitude, and Practice Towards Antibiotics Use Among Medical Sector Final-Year Students in Egypt.

**Journal name:** Medical Science Educator

**Author name:** Nourhan M. Emera

**Email address:** Nourhan.mo.emera@pharma.cu.edu.eg

**Appendix 5 Practice towards antibiotics among participating students**

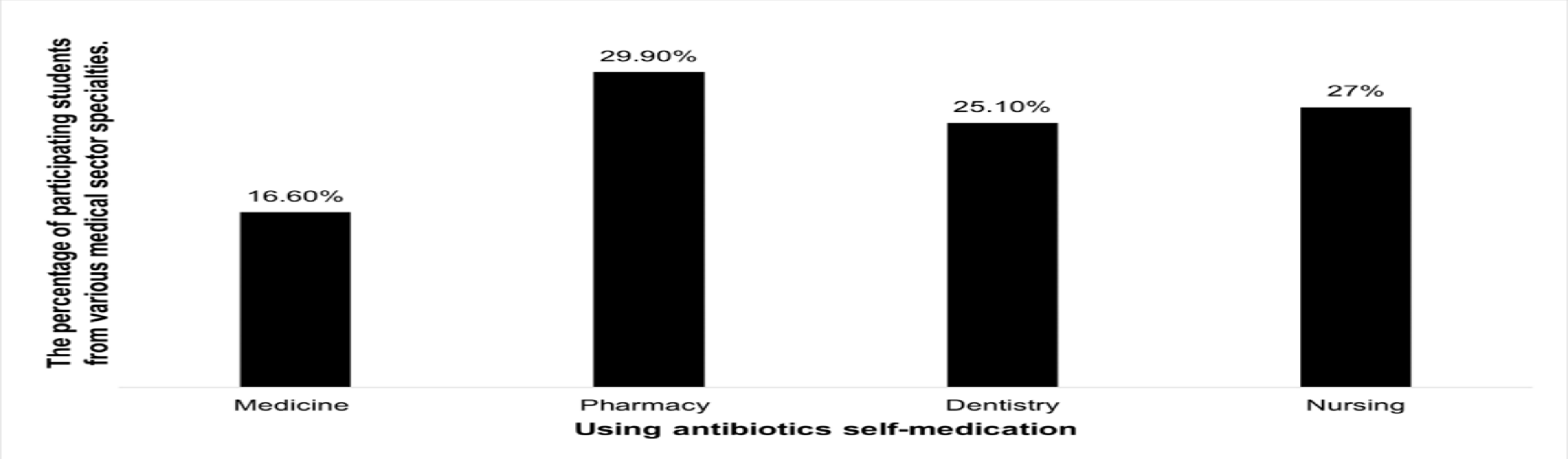


**Figure 1. The frequency (Expressed as percentages) of antibiotics self-medication (ABSM) among students of different specialties. (N=1250).**

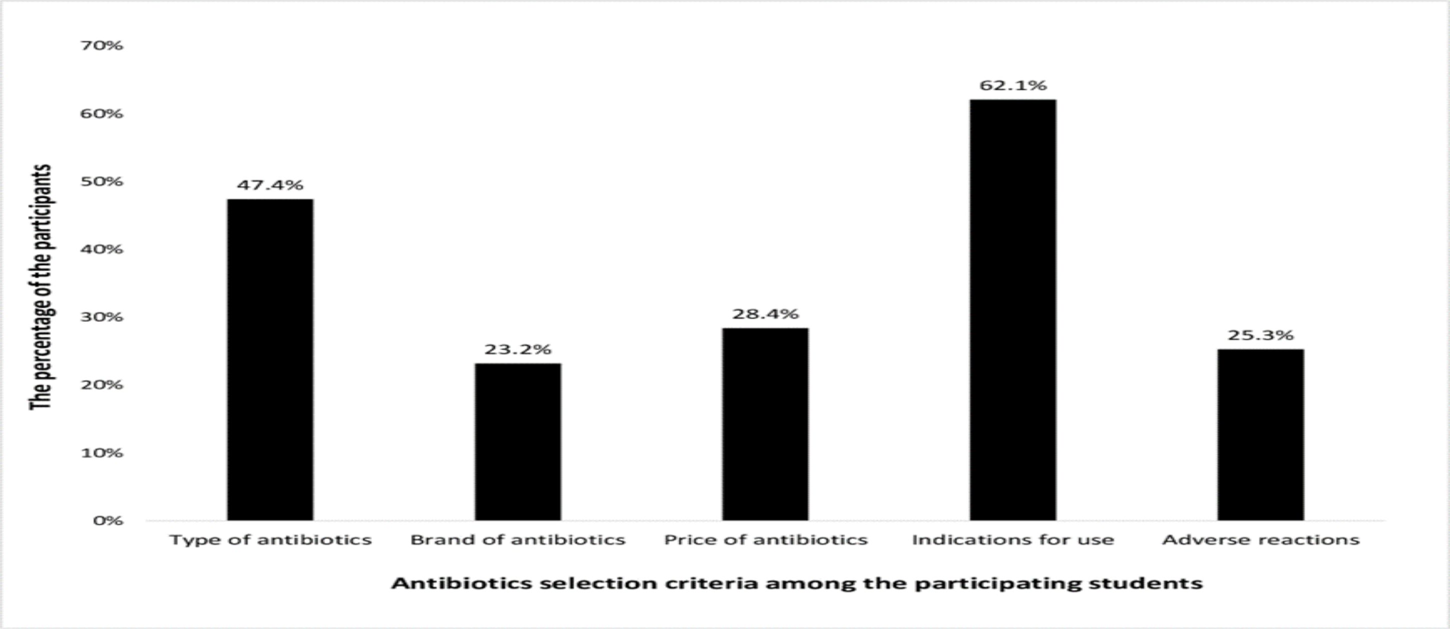


**Figure 2. Antibiotics selection criteria among the 1250 surveyed students (Expressed as percentages).**

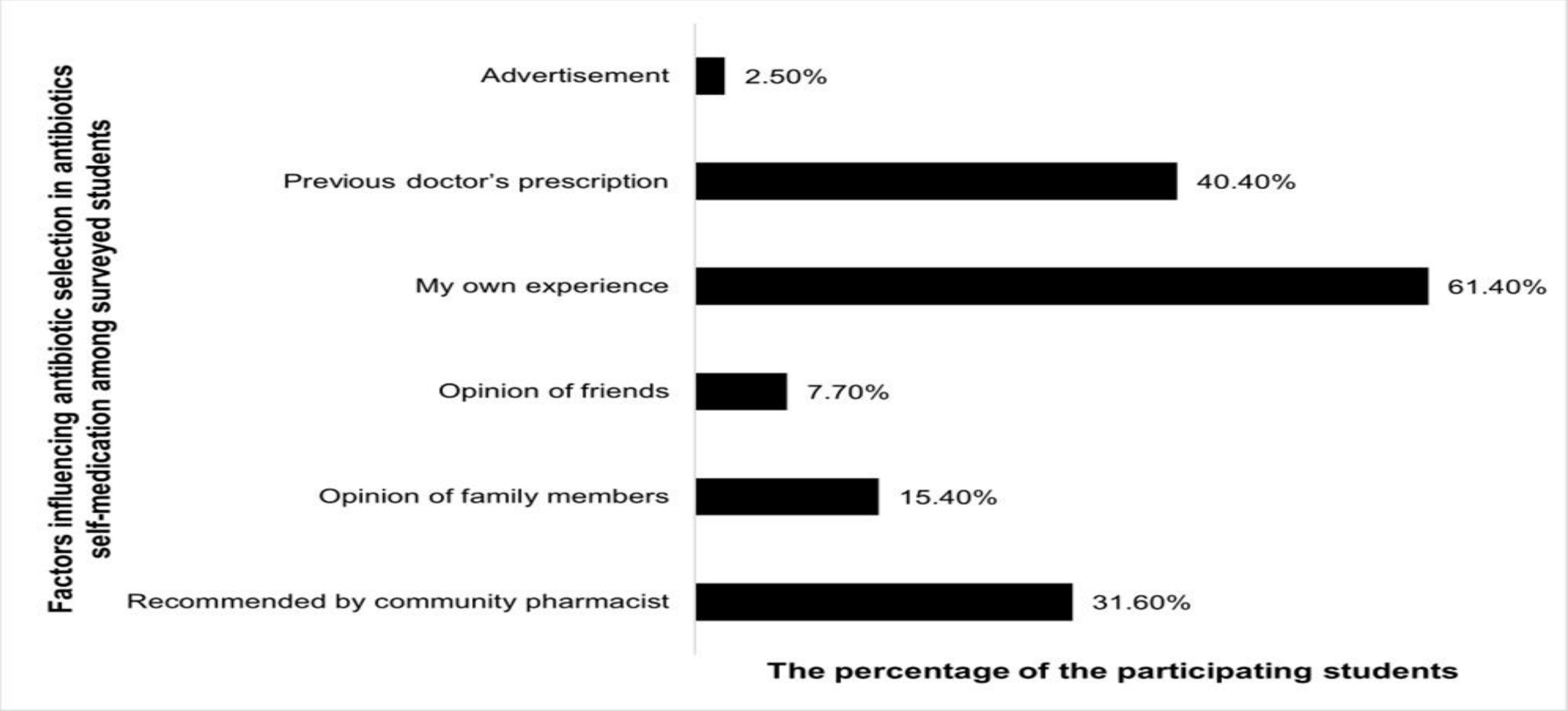


**Figure 3. Information resources on antibiotics among the 1250 surveyed students (Expressed as percentages)**

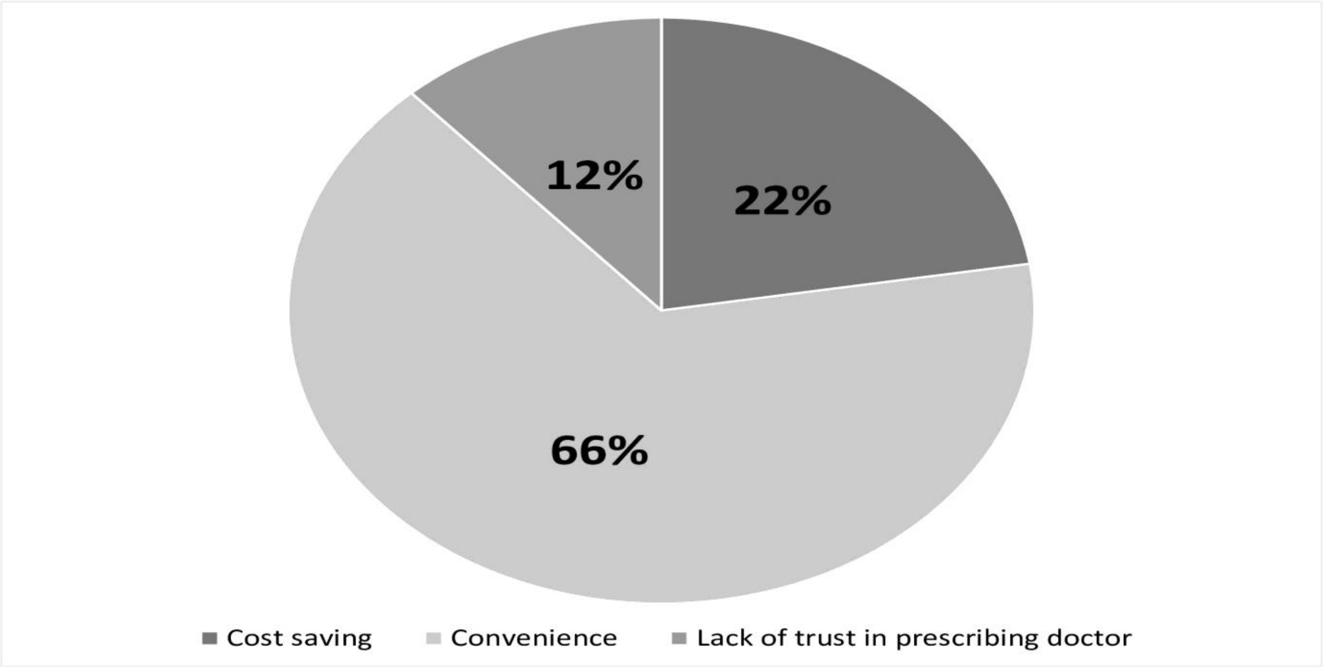


**Figure 4. Reasons for antibiotics self-medication as specified by the 1250 surveyed students (Expressed as percentage).**

**Table 4.** The practice of the surveyed students about antibiotics (presented as response frequencies (%)).

| Questions of practices towards antibiotic use and its resistance (Response) | Total (N= 1250) | MS (N = 217) | PS (N = 388) | DS (N= 291) | NS (N = 354) | P |
|---|---|---|---|---|---|---|
| **Do you consult a doctor before starting antibiotics? (Yes)** | 871 (69.7) | 175 (80.6) | 245 (63.1) | 201 (69.1) | 250 (70.6) | <0.001 [a] |
| **Pairwise comparisons (P)** | $P_{MP}$ <0.001 [a] | $P_{MD}$ = 0.011 [a] | $P_{MN}$ = 0.021 [a] | $P_{PD}$ = 0.274 | $P_{PN}$ < 0.013 [a] | $P_{PN}$ = 0.160 |
| **P.5. The Doctor prescribes a course of antibiotics for you. After taking 2–3 doses you start feeling better** | | | | | | |
| **A.Do you stop taking the further treatment?(No)** | 1017 (81.4) | 188(86.6) | 323(83.2) | 244(83.8) | 262(74.0) | = 0.001 [a] |
| **Pairwise comparisons (P)** | $P_{MP}$ =0.271 | $P_{MD}$ = 0.427 | $P_{MN}$ < 0.001 [a] | $P_{PD}$ = 0.976 | $P_{PN}$ < 0.005 [a] | $P_{PN}$ < 0.006 [a] |
| **B. Do you complete the full course of treatment? (Yes)** | 1038(83.0) | 188(86.6) | 337(86.9) | 240(82.5) | 273(77.1) | = 0.002 [a] |
| **Pairwise comparisons (P)** | $P_{MP}$ < 0.001 [a] | $P_{MD}$ = 0.091 | $P_{MN}$ = 0.502 | $P_{PD}$ = 0.006 [a] | $P_{PN}$ < 0.001 [a] | $P_{PN}$ = 0.533 |
| **C. Do you save the remaining antibiotics for the next time you get sick? (No)** | 641 (51.3) | 96 (44.2) | 230 (61.1) | 146 (50.2) | 162 (45.8) | <0.001 [a] |
| **Pairwise comparisons (P)** | $P_{MP}$ < 0.001 [a] | $P_{MD}$ = 0.091 | $P_{MN}$ = 0.502 | $P_{PD}$ = 0.006 [a] | $P_{PN}$ < 0.001 [a] | $P_{PN}$ = 0.533 |

| | | | | | | |
|---|---|---|---|---|---|---|
| **D. Do you discard the remaining antibiotics?** | 795 (63.6) | 152 (70.0) | 230(59.3) | 204(70.1) | 209(59.0) | <0.001 [a] |
| **Pairwise comparisons (P)** | $P_{MP}$ < 0.001 [a] | $P_{MD}$ = 0.091 | $P_{MN}$ = 0.502 | $P_{PD}$ = 0.006 [a] | $P_{PN}$ < 0.001 [a] | $P_{PN}$ = 0.533 |
| **E. Do you give the leftover antibiotics to your friend/family if they get sick? (No)** | 811 (64.9) | 138 (63.6) | 275 (70.9) | 193 (66.3) | 205 (57.9) | 0.006 [a] |
| **Pairwise comparisons (P)** | $P_{MP}$ = 0.178 | $P_{MD}$ = 0.465 | $P_{MN}$ = 0.177 | $P_{PD}$ = 0.200 | $P_{PN}$ < 0.001 [a] | $P_{PN}$ = 0.039 |
| **P.6. If you experience side effects of antibiotics:** | | | | | | |
| **Do you stop taking the antibiotic without consulting a doctor or pharmacist? (Yes)** | 385 (30.8) | 67 (30.9) | 97 (25.0) | 86 (29.6) | 135 (38.1) | <0.001 [a] |
| **Pairwise comparisons (P)** | $P_{MP}$ < 0.043 [a] | $P_{MD}$ = 0.233 | $P_{MN}$ < 0.001 [a] | $P_{PD}$ = 0.015 [a] | $P_{PN}$ < 0.001 [a] | $P_{PN}$ < 0.001 [a] |
| **P.7. If you took the wrong antibiotics:** | | | | | | |
| **Do you visit the doctor immediately? (Yes)** | 808 (64.6) | 126 (58.1) | 252 (64.9) | 177 (60.8) | 253 (71.5) | 0.013 [a] |
| **Pairwise comparisons (P)** | $P_{MP}$ = 0.130 | $P_{MD}$ = 0.374 | $P_{MN}$ = 0.001 [a] | $P_{PD}$ = 0.467 | $P_{PN}$ = 0.148 | $P_{PN}$ = 0.017 [a] |
| **P.8. You read the instructions in the package insert & check the expiry date before taking antibiotics. (Yes)** | 1096(87.7) | 186(85.7) | 331(85.3) | 266(91.4) | 313(88.4) | =0.127 |

Abbreviations: MS = Medicine students, PS= Pharmacy students, DS = Dentistry students, NS = Nursing students, N: Number of students. Comparison between groups and pairwise comparisons were used with the Chi-square Fisher Exact test (the p-value on the right column of the table). P: p-value at level of significance < 0.05, $P_{MP}$: p-value for comparing medicine and pharmacy, $P_{MD}$: p-value for comparing medicine and dentistry, $P_{MN}$: p-value for comparing medicine and nursing, $P_{PD}$: p-value for comparing pharmacy and dentistry, $P_{PN}$: p-value for comparing pharmacy and nursing, $P_{DN}$: p-value for comparing dentistry and nursing. [a]: statistically significant.
