# Supplementary material for: Knowledge, Attitude, and Practice Towards Antibiotics Use Among Medical Sector Final-Year Students in Egypt
Source: Med Sci Educ. 2024 Aug 2;34(6):1369–79. doi: 10.1007/s40670-024-02117-6 (PMC11698705; doi:10.1007/s40670-024-02117-6)
Supplement: Supplementary file 6 — Supplementary file6 (PDF 474 KB) [file 40670_2024_2117_MOESM6_ESM.pdf]

Article title: Knowledge, Attitude, and Practice Towards Antibiotics Use Among Medical Sector Final-Year Students in Egypt.

Journal name: Medical Science Educator

Author name: Nourhan M. Emera

Email address: Nourhan.mo.emera@pharma.cu.edu.eg

Appendix 6. The results of all questions about the Sources of information about antibiotics and the topics taught in the medical curriculum.

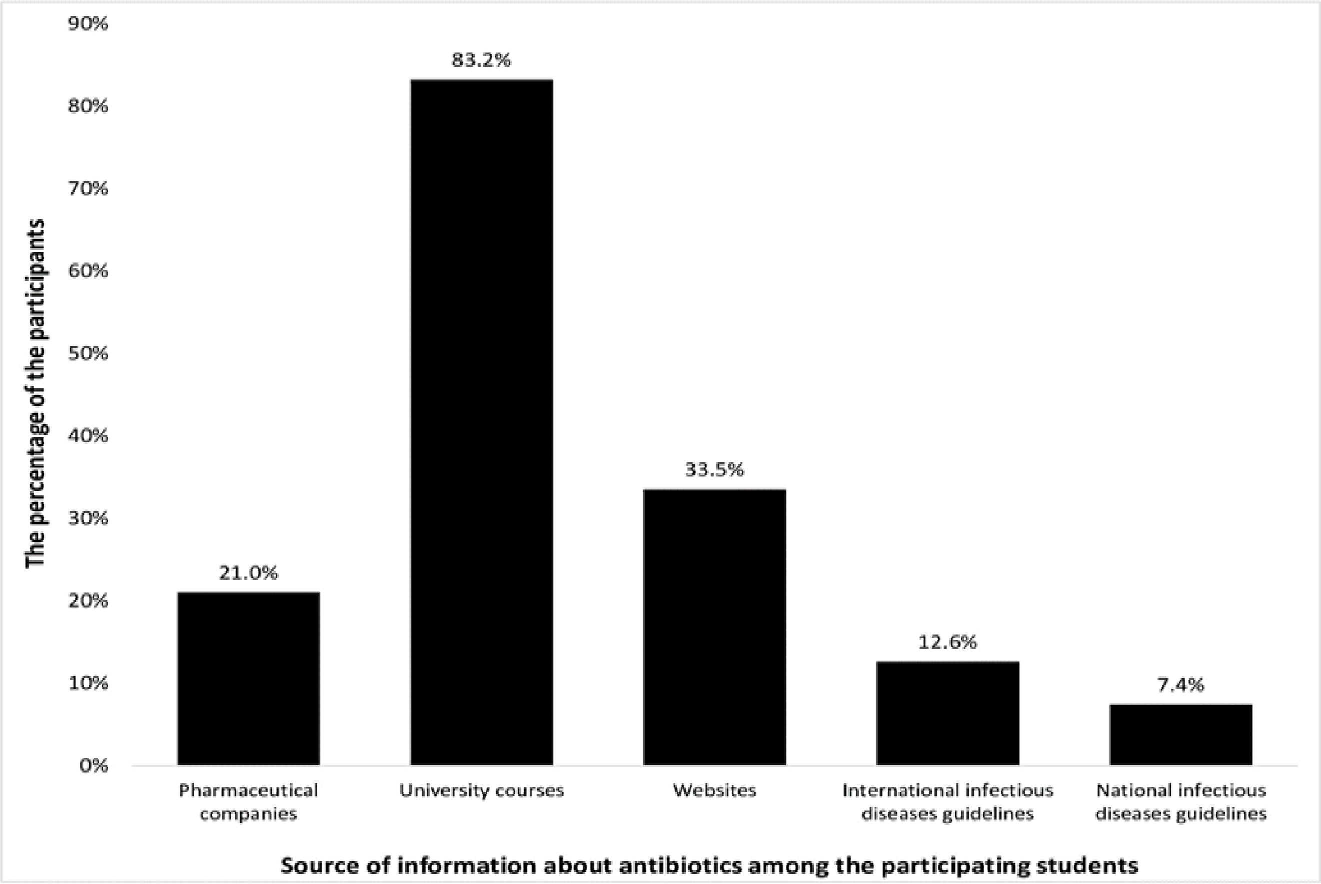

Figure 1. Source of information about antibiotics among the 1250 surveyed students (Expressed as percentages).

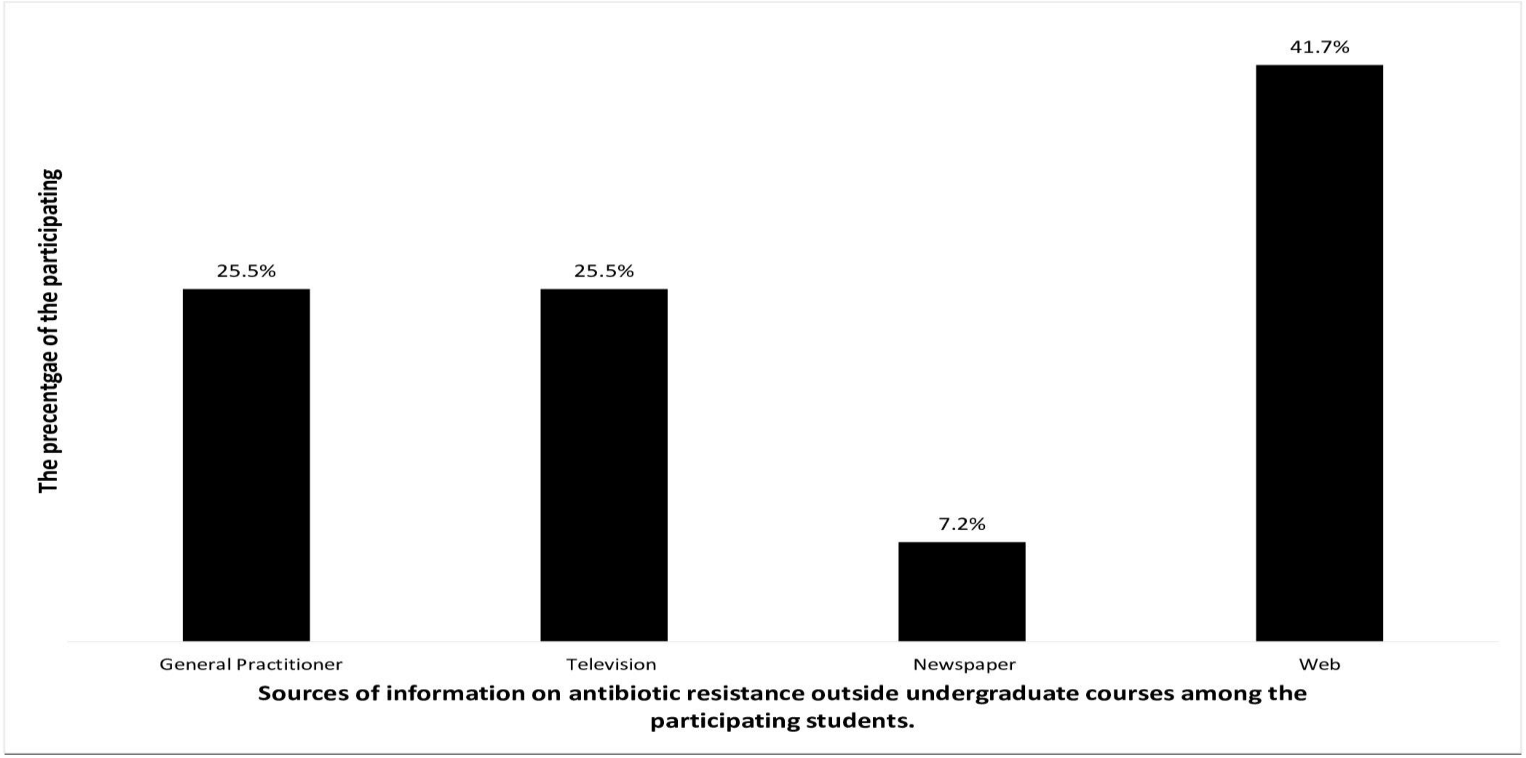

Figure 1. Percentage of surveyed students (N=1250) from different medical specialties reporting sources of information on antibiotic resistance outside undergraduate courses.

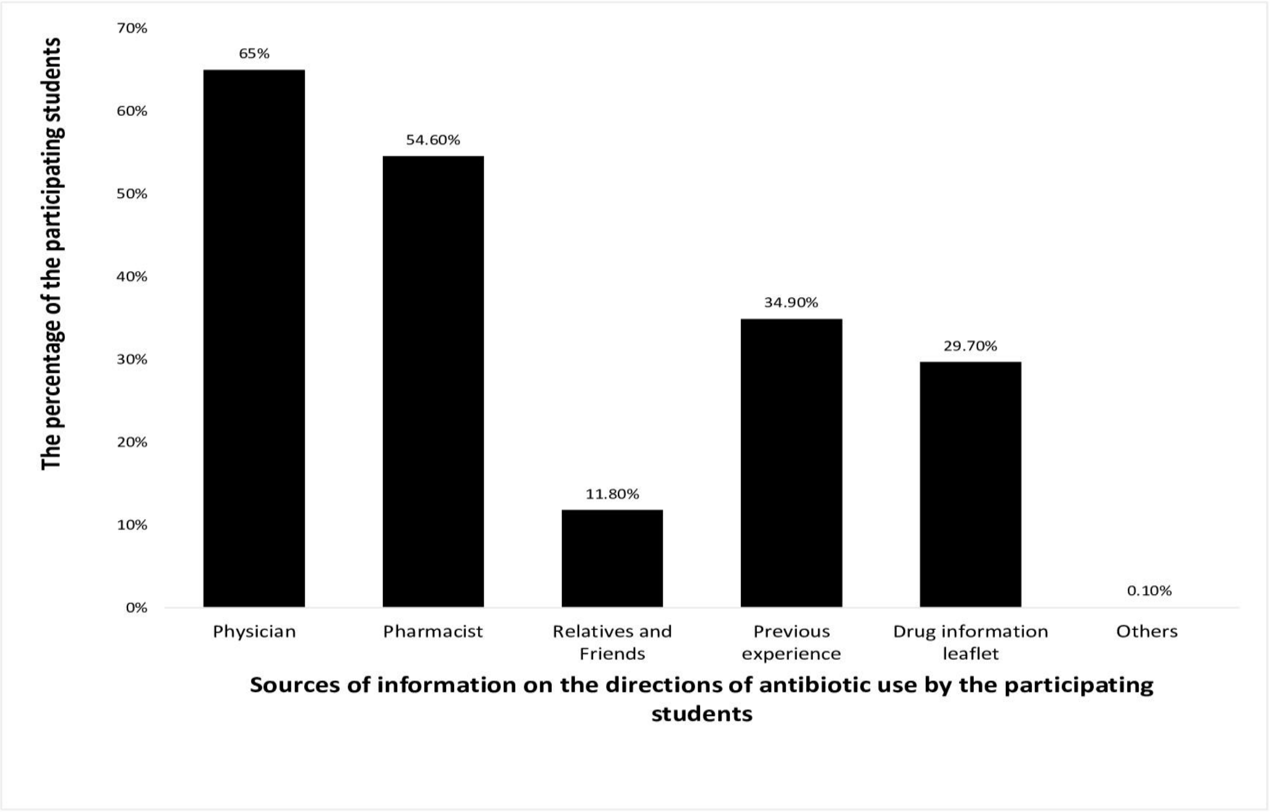

Figure3. Percentage of surveyed students (N=1250) from different medical specialties reporting sources of information on the directions of antibiotic use.

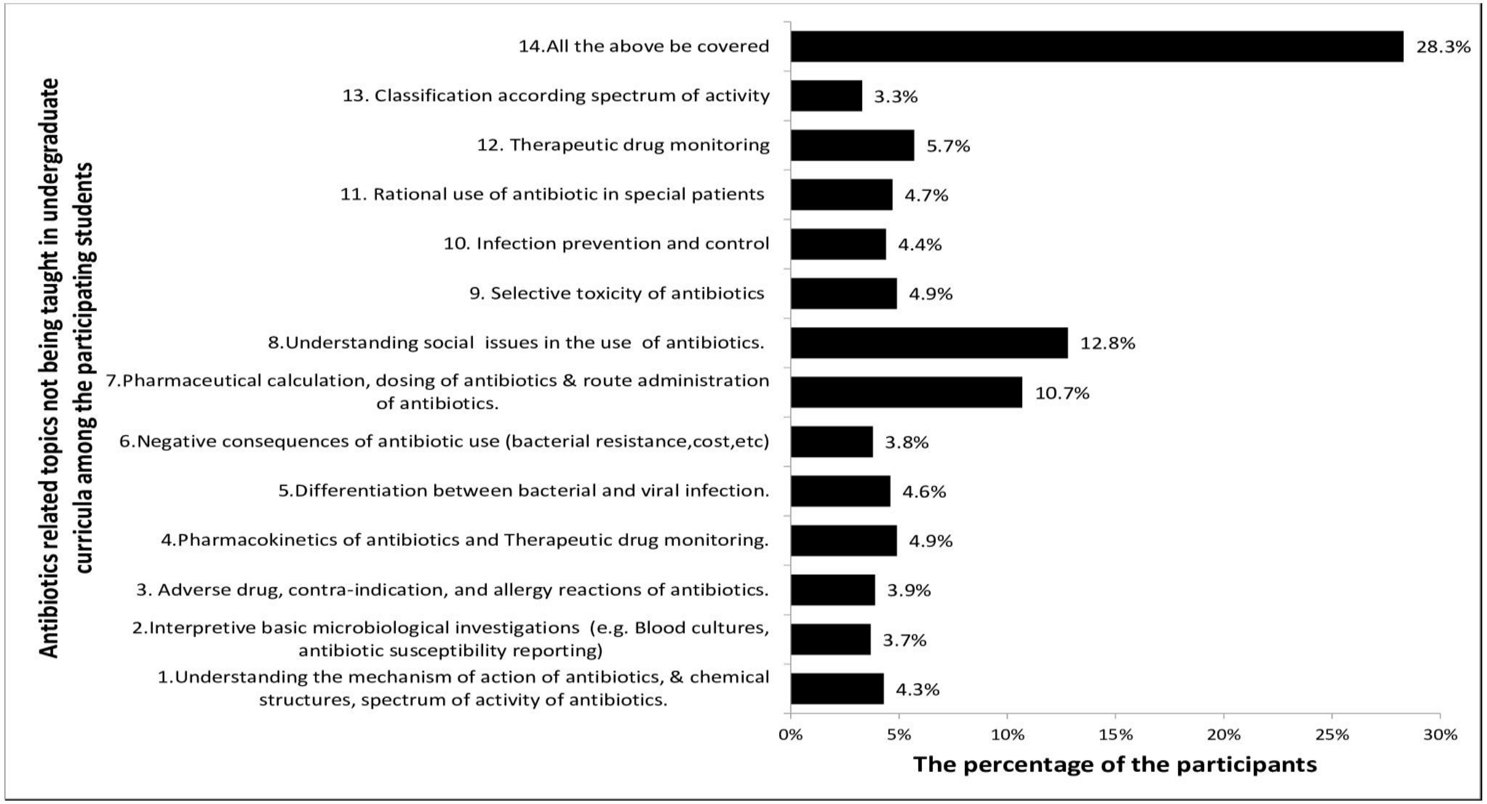

Figure 2. Percentage of surveyed students (N=1250) from different medical specialties indicating insufficient coverage of antibiotics-related topics in undergraduate curricula.

Table 5. Comparison between the study groups about antibiotics in the medical curriculum (Expressed as number (%)). (N=1250)

Questions about the topics taught in the medical curriculum (Response)	Students Specialties				
	Total (N = 1250)	MS (N = 217)	PS (N = 388)	DS (N = 291)	NS (N = 354)
T.2.1. Have you ever heard of antibiotic resistance outside your undergraduate courses? (Yes)	895 (71.6)	160 (73.7)	289 (74.5)	210 (72.2)	236 (66.7)
T.4.1 Have you received any type of training about antibiotics? (No)	1148 (91.8)	211 (97.2)	327 (94.3)	284 (97.6)	326 (92.1)
T.5.1. Do you think that you need more education about an antibiotic? (Yes)	611 (48.9)	104 (47.9)	190 (49)	161 (55.3)	156 (44.1)

N= Number of students
